# Development and external validation of a risk prediction score (DASHI) for cardiovascular events following acute respiratory infections: derivation and validation retrospective cohort study

**DOI:** 10.1016/j.eclinm.2025.103273

**Published:** 2025-06-02

**Authors:** Joseph J. Lee, Constantinos Koshiaris, Cynthia Wright-Drakesmith, Jennifer A. Davidson, Charlotte Warren-Gash, F.D. Richard Hobbs, James P. Sheppard

**Affiliations:** a Nuffield Department of Primary Care Health Sciences, University of Oxford, UK; b Faculty of Epidemiology & Population Health, London School of Hygiene & Tropical Medicine, Keppel Street, London WC1E 7HT, UK; c Department of Primary Care and Population Health, University of Nicosia Medical School, Engomi, Nicosia CY-2414, Cyprus

**Keywords:** Respiratory infection, Cardiovascular events, Prediction, Validation, Primary care

## Abstract

**Background:**

Acute respiratory infections increase the short-term risk of myocardial infarction (MI) and stroke in primary care patients. Clinical guidelines for acute respiratory infections in primary care do not consider the risk of cardiovascular events, and CVD risk prediction tools target long-term risk. We aimed to develop and validate a prediction tool for the risk of cardiovascular disease events within 28-days of acute respiratory infection.

**Methods:**

The design was a retrospective cohort study using two different databases of routinely collected data from electronic health records from January 1999 to December 2019. We used Clinical Practice Research Datalink (CPRD) Aurum data to derive models, and CPRD GOLD data from a different population for external validation. This data is from UK primary care, with data linkage to Hospital Episode Statistics, Office of National Statistics mortality data, and Index of Multiple Deprivation data. Participants were patients aged 40 years or older with no history of cardiovascular events, and a first diagnosis with acute respiratory infection. The outcome was a composite of new diagnoses of myocardial ischaemia (myocardial infarction, angina, acute coronary syndromes, or ischaemic cardiomyopathy), stroke or transient ischaemic attack, or deaths with these diagnoses, within 28 days of presentation with an acute respiratory infection. We derived a list of 57 potential predictors based on prior studies and asked clinical experts to rank them. We derived two logistic regression models, one with the top ranked variables, and another including additional lower ranked variables. We derived a clinical prediction score from the most parsimonious logistic regression model. We validated each model and the score in the external dataset using C statistics, calibration plots, and expected to observed ratios. We examined clinical utility using decision curve analysis.

**Findings:**

The derivation cohort comprised 3.8 million patients with an acute respiratory infection (mean age 56.5 years, (SD 13.7); 57.7% female), of whom 11,996 had a subsequent cardiovascular outcome (0.3%). The validation cohort included 2.6 million patients (mean age 56.7 years, SD 13.6, 58.0% female), of whom 6868 (0.3%) had a subsequent cardiovascular outcome. The DASHI score comprised five clinical variables: Diabetes (1 point, yes/no), Age (40–59, 0 points; 60–79, 2 points; 80+, 4 points), current Smoking (1 point, yes/no), Heart failure (1 point, yes/no), and Infection diagnosis (Upper Respiratory Tract Infection–0 points. Lower Respiratory Tract Infection (LRTI)–1 point, or LRTI with a pneumonia diagnosis—4 points). Upon external validation, each model and the score showed similar performance. The score showed good discrimination (AUC 0.85, IQR 0.848–0.849) and calibration with an expected to observed ratio of 0.85 (IQR 0.85–0.85).

**Interpretation:**

The DASHI score allows primary care clinicians to estimate the risk of cardiovascular complications within 28 days in patients with acute respiratory infections.

**Funding:**

This research was funded in part by the 10.13039/100010269Wellcome Trust [211182/Z/18/Z] and NIHR [NIHR300738]. For the purpose of open access, the author has applied a CC BY public copyright licence to any Author Accepted Manuscript version arising from this submission. The views expressed are those of the authors and not necessarily those of the NIHR or the Department of Health and Social Care.

**Research in context**

**Evidence before this study**

We searched Medline from inception to August 9th 2024 with the terms ‘Acute respiratory infection’ AND ‘myocardial infarction OR stroke’ AND ‘prediction’. This returned 419 results. There were studies describing the association between infections and myocardial infarction and stroke, but none attempting to predict the risk of CVD events after acute respiratory infection.

**Added value of this study**

We have developed and validated the DASHI score to predict the risk of a primary cardiovascular event in the 28 days following acute respiratory infection, using data from 6.4 million patients. The DASHI score comprises five clinical variables: Diabetes, Age, Smoking, Heart failure, and Infection type.

**Implications of all the available evidence**

Respiratory infections increase the risk of cardiovascular events. The absolute risk of primary CVD events is low. The DASHI score can help predict this risk, but the clinical utility is limited by low prevalence.

## Introduction

Acute respiratory infections increase the short-term risk of myocardial infarction (MI) and stroke in primary care patients.^1^ Acute respiratory infection approximately quadruples the background risk of an acute cardiovascular event occurring in the following four weeks.^2^, ^3^, ^4^, ^5^, ^6^, ^7^ The risk is highest in the first few days. This relationship between cardiovascular disease (CVD) events and infections is well established.^4^

Post-infection CVD events are not restricted to a particular infectious organism. Considerable epidemiological evidence relates to undifferentiated acute respiratory infections, and there is evidence relating to a variety of organisms.^5^ COVID-19 also increases short-term CVD event risk, but most of the existing evidence predates this pandemic.^3^ Influenza and pneumococcal infections are both associated with CVD events, but other organisms are also implicated in the seasonality of CVD.^8^ There is evidence that preventing infection mitigates CVD risk, including trials that show influenza vaccine reduces CVD events and observational evidence of the same association for pneumococcal vaccines.^9^, ^10^, ^11^, ^12^

Whilst CVD events appear to be a serious complication of acute respiratory infection, at present clinical guidelines for acute respiratory infections in primary care do not consider the risk of CVD events.^1^^,^^13^ There may be an opportunity for intensifying CVD prevention during infections and for the weeks following infection. Informing patients of their risk could help conversations about primary prevention, safety netting, and future vaccinations.

There are also precedents for short-term risk modification at peaks of CVD event risk. High-risk transient ischaemic attack (TIA) or minor stroke also herald a short high-risk period for stroke.^14^ Long-term antiplatelets are not recommended for primary prevention, because the risk of bleeding outweighs the benefits.^1^ However, following TIA, dual antiplatelet therapy, plus a statin, for a few weeks reduces the risk of stroke without an unacceptable increase in the risk of bleeding.^15^ Investigating analogous strategies in infections is a research priority. To develop such evidence would first require a validated method to identify people with infections who are at increased risk of CVD events.

There are many CVD risk prediction models, but these typically aim to predict long-term or lifetime risk based on demographic and lifestyle factors, and chronic health conditions. None considers fluctuating short-term increases in CVD risk associated with acute respiratory infections.^16^ We therefore aimed to derive, and externally validate, a parsimonious prediction model for acute respiratory-infection related CVD event risk.

## Methods

### Aim

Our aim was to produce a clinically useful prediction tool for CVD event risk for people with acute respiratory infection.

### Objectives

Our first objective was to derive a simple statistical prediction model; model one, comprising a small number of clinical variables. Objective two was to derive another more comprehensive model; model two, including all the predictors from model one with extra predictors. Our third objective was to externally validate models one and two. We then derived and validated a points-based clinical prediction score, and compared the score and statistical models with decision curve analysis.

### Data source

We used two different retrospective observational cohorts to derive and validate the statistical models and prediction score.

The cohorts were derived from data in the Clinical Practice Research Datalink (CPRD) GOLD and Aurum databases. These are databases of UK primary care records from different electronic clinical records systems. These data were linked to Hospital Episode Statistics (HES) and Office of National Statistics (ONS) data. These were Index of Multiple Deprivation (IMD–an official measure of deprivation in England) and ONS mortality data from death certificates (Supplementary methods). The collection and classification of sex was out of our control, as these are routinely collected data provided by CPRD.

### Ethics

The CPRD Independent Scientific Advisory Committee (ISAC) approved the pre-specified protocol (21_000380). This study was undertaken under CRPD’s generic ethics approval for observational studies that make use of anonymised data and have prior approval of the ISAC (Multiple Research Ethics Committee ref. 05/MRE04/87). CPRD has ethics approval from the Health Research Authority to support research using anonymised patient data under which this study was deemed exempt from obtaining informed consent (National Research Ethics Service Committee (NRES) ethics approval 21/EM/0265).

### Population

The index date was the date of first diagnosis with acute respiratory tract infection (RTI) after age 40. There was no upper age limit. We followed patients for 28 days from the index date.

We chose to include people over the age of 40 years because of a low risk of events in people under this age. There is also precedent for using this cutoff—it was used in QRISK2–the clinical model most used for stratifying the primary prevention population in the UK.^17^ We used 28 days as this period has been consistently associated with an increase in risk following infections in primary care.^5^

It is possible that some patients had prior CVD, but this was not recorded. We suspect this would be minimal, as this misclassification would require no record in either primary care or secondary care records of an important event. This would reflect the information available to the GP at use of the model, and so clinical reality.

We identified acute respiratory infections by codes in the primary care record (https://github.com/Protocols-For-Research/CPRD-codes-CVD-infection-risk). We excluded infections likely to be chronic. We used three mutually exclusive categories of acute respiratory tract infection (upper, lower, and lower with pneumonia), plus influenza-like illnesses (ILI) which was not exclusive to the other three categories. We defined upper respiratory tract infections (URTIs) to include anatomical sites at or above the larynx. Lower respiratory tract infections (LRTIs) included any codes for acute infections below the larynx. Infective exacerbations of chronic obstructive pulmonary disease (COPD) we included as LRTIs. We defined LRTI with a diagnosis of pneumonia (pneumonia) as a LRTI with one or more codes identifying pneumonia. If a patient had codes for more than one category we allocated the more severe; pneumonia trumped LRTI and LRTI trumped URTI, making these mutually exclusive. Whilst we were able to identify codes for ILI, these overlapped the other categories. Some of the codes for influenza and ILI don’t indicate an anatomical site; other codes specify influenza causing an URTI or a pneumonia.

We excluded patients with a history of CVD events (defined as below) and those with less than 1 year at a practice, to avoid recording old CVD diagnoses as new, which could happen when patients register at CPRD practices for the first time.^18^

### Outcome

The outcome was a composite of cardiovascular codes, occurring within 28 days of a diagnosis of acute respiratory infection. It included new myocardial ischaemia (myocardial infarction, angina, acute coronary syndromes, or ischaemic cardiomyopathy), new cerebrovascular events (stroke, and TIA), and deaths from these causes.

We used primary and secondary care clinical codes, and ONS mortality records to identify outcomes.

### Outcome details

The outcome is a combination of cardiovascular events, within 28 days of the acute respiratory infection. This was a composite of new diagnoses of myocardial ischaemia, stroke, TIA, or deaths from these. We thought this approach came close to the ‘four point Major Adverse Cardiovascular Event (MACE)’ outcome used in trials of antidiabetic medications and largely caused by atherosclerosis.^19^ We could not use ischaemic stroke as an outcome, because stroke type is poorly differentiated in CPRD data.^20^ We did not include generic ‘heart failure’ diagnosis in the outcome, as it may be due to aetiology other than ischaemia, though we included specifically coded new ischaemic cardiomyopathy. We did not include peripheral vascular events such as limb ischaemia or claudication in the outcome. Instead, we included markers of peripheral arterial disease in the candidate predictors. Similarly, we did not include other chronic cardiac conditions in the outcome as they would not be triggered by a RTI (valve disease, congenital disease etc) and so included chronic heart disease as a predictor instead.

As we used routinely collected datasets there was no blinding of the outcome assessors—and no risk of this study biasing these clinicians’ historic assessments.

The outcome we derived from CPRD, ONS mortality and HES datasets. Hospital Episode Statistics data includes diagnosis data and procedure data.

CPRD recommend the use of search strategies rather than specific codelists.^21^ We searched CPRD code files using Stata. We aimed to make code lists specific, rather than over-sensitive. As an example, we did not use codes for referrals to chest pain or TIA clinics in the outcome, as many of these patients go on have CVD events ruled out.

### Codes employed

CPRD codes do not map directly to ICD codes, and so we had to develop our own codelists. The type and site of infection is very likely prone to misclassification. In the UK primary care situation diagnosis is clinical, without test. For example, clinical risk scores for influenza have diagnostic performance that is moderately predictive.^22^ The imperfect data reflects the information that the clinicians would have and is likely a proxy for severity of infection. Misclassification would bias towards a lack of association between the misclassified variables and CVD, but despite this we have shown a strong association with outcomes, so it does not appear to have greatly impacted the validation results.

The CPRD codes we used can be found here: https://github.com/Protocols-For-Research/CPRD-codes-CVD-infection-risk. The ICD and OPCS codes used for the outcome are in the Supplementary materials.

### Predictors

We identified predictors from previous publications and expert clinical review. We identified 54 clinical variables associated with CVD, severe acute respiratory infections, or both. Clinical variables included demographics, medications, laboratory tests and physical measurements. To prioritise variables for inclusion four general practitioners with an interest in cardiology assessed their perceived importance. The clinicians ranked the clinical variables in order of importance considering both association with CVD, and the accuracy of coding (Supplementary appendix).

In the absence of previously published models, we generated two statistical models to assess the trade-off between complexity and performance. We then chose a model to develop a risk score.

We included infection type in both models. There were three exclusive categories of infection type; Upper Respiratory Tract Infection (URTI), Lower Respiratory Tract Infection (LRTI), and LRTI with a diagnosis of pneumonia. We also included an additional binary variable for influenza diagnosis, which was not exclusive of the other infection types.

In model one we also included the top clinical variables as ranked by the clinical experts. Model two included additional clinical variables from further down the ranking and the lowest ranked were not included.

### Predictor selection

We included infection type in all models. Four General Practitioners (GPs) with special interest in cardiology helped rank the other clinical variables. We asked them to rank the relevance of 54 variables^d^ from minus three to three. We asked them to consider the completeness of coding as well as clinical relevance. We standardised each GP’s ratings into Z scores to express the relative importance they gave each variable on the same scale, with a mean of zero and a standard deviation of one. We then combined these scaled scores by arithmetic mean across GPs to give a mean Z score. We used this overall mean ranking to order the variables. Model one included those variables with a mean Z score >1 and model two included variables with a mean Z score >0. Variables with a mean Z score of 0 or less were not included in models.

By ‘clinical variable’, we mean a diagnosis, demographic, or test, as ranked by the experts, rather than a term in a statistical model. For example, smoking status was a single clinical variable for clinicians to rank but is represented in the statistical models as multiple categorical variables. These represent people who have never smoked, ex-smokers, light smokers (<10 cigarettes per day), moderate smokers (10–19 cigarettes per day), heavy smokers (20+ cigarettes per day), and those who smoke an unquantified amount.

We derived all the predictors from the clinical records and linked datasets (we did not collect other data). We extracted covariates from the clinical record before the index date. We had to define a relevant time window before the infection to search for relevant codes. We used different time windows for different variables. For cholesterol to HDL ratio, BMI, and systolic blood pressure, we used the most recent record in the five years before the index date. Cancers also had a five-year limit. We took codes for other diagnoses, and family history, from the entire record prior to the index date. In order to better identify ex-smokers, and the amount people smoked, we used the two most recent smoking records. We defined smoking categories as: never smoked, ex-smoker, light (<10 cigarettes per day), medium (10–19 cigarettes per day), heavy (20+ cigarettes per day) and amount unknown.

### Refinement of variables

#### Respiratory infections

The coding systems shaped the respiratory infection diagnosis variable. We were able to classify some respiratory infections into exclusive groups: upper respiratory tract infection (URTI), lower respiratory tract infection (LRTI), and LRTI with pneumonia diagnosis are categories without overlap codes suggesting infections from the trachea up we counted as URTI, those below this were LRTIs, and specific codes for pneumonia, or it’s complications, were used for pneumonia. Influenza was not an exclusive category—there are codes for influenza without specifying severity or site, but other codes for influenza associated with URTIs, LRTIs, and pneumonia. Influenza is therefore not an exclusive category. We classed exacerbations of Chronic Obstructive Pulmonary Disease (COPD) as lower respiratory tract infection, unless there were codes indicating the exacerbation was a pneumonia. To avoid collinearity COPD was not included as a separate variable.

#### Covariates

We refined, combined and dropped some of the clinical categories after the clinical experts had prioritized them. These were COPD, hypertension, dementia, diabetes subtypes, non-steroidal anti-inflammatories, erectile dysfunction, chronic kidney disease, peripheral vascular disease, cancer subtypes, family history of CVD, and vaccination status.

The top ranked variables for model one, before refinement into the final model were: Age, heart failure, diabetes, smoking status, chronic kidney disease, peripheral vascular disease and COPD. For initial model two, we included those in model one plus variables further down the ranking. The ranking continued: systolic blood pressure, sex, cholesterol to HDL ratio, BMI, atrial arrhythmias, dementia, anticoagulants, NSAIDS, antiplatelets, antihypertensives, rheumatoid arthritis, statin use, platelets, CRP, erectile dysfunction, other chronic heart diseases (including valve disease, congenital disease), IMD decile, haematological cancers, solid cancers, family history of CVD in first degree relative less than 60 years of age, and Pneumococcal vaccine.

We first defined family history of CVD as an event in a first-degree relative aged less than sixty, reflecting the definition used by QRISK3.^17^ Unfortunately, the coding systems do not include codes for this. Instead, we defined a high-risk family history as being a CVD aged less than 65 years if the first-degree relative was female, and fifty-five if male.

We simplified variables by combining rare and overlapping categories. We combined diabetes mellitus type one (which was rare), with type two diabetes and diabetes of other and unspecified types. We did not include the strongly correlated ‘glucose lowering medications’. Haematological cancers were uncommon, so we combined them with solid cancers. We also combined peripheral vascular disease with chronic kidney disease and erectile dysfunction. These three predictors were sparse, and are diagnoses that can have common cause in underlying atherosclerosis. Over the course of the study, the UK was introducing various pneumococcal vaccines against different serotypes, and for different populations (for over 65’s from 2003 for example), so we did not include pneumococcal vaccination as a predictor.^23^ Dementia was the only variable excluded on the basis of odds ratio, which was 1.00. No variable selection methods were used that rely on p values because we considered it likely the size of the dataset would lead to very small p values for every variable.

#### Statistics

We calculated baseline descriptive statistics in both cohorts including by outcome status. We estimated means and standard deviations for continuous variables, and numbers and percentages for categorical variables.

We calculated sample size calculations for model derivation before study protocol approval using methods by Riley et al.^24^ We based the calculation on preliminary counts in CPRD Gold data; this gave us a conservative outcome prevalence of 0.089%. We aimed for a global shrinkage factor of >0.995. We assumed a maximum of 50 candidate variables (including transformations and interactions). We also checked we would meet the criteria of an absolute difference of <0.05 in apparent and adjusted Nagelkerke’s R^2^, and a margin of error in outcome proportion estimates for null model <0.05. We calculated 61,198 patients would be enough to achieve these criteria.

We also performed a post-hoc sample size calculation for model validation. Derivation sample sizes tend to need to be larger than validation samples, so we relied on this in the protocol. In addition, at the point of writing the protocol we did not have the results from the derivation to use for the calculation. We used the user written Stata package pmvalsampsize, using estimates from internal calibration of the DASHI score.^25^ We specified a prevalence of 0.3%, a C statistic of 0.84, and a calibration slope of 1.07. We modelled the distribution of log predicted probabilities with the results from the development data: a skewed normal distribution with a mean of −7.074499 and a variance of 1.030443, skewness of 1 and kurtosis of 4. This returned a result of 115,597 patients being required, with estimating the calibration slope as the most data hungry calculation (observed to expected ratio required 5510 and the C statistic 20,525). Our dataset exceeded these numbers.

#### Missing data

We used the same methods in each cohort. If there were no codes for binary variables prior to the index date, we assumed the disease or prescription was absent.

For continuous and categorical variables with missing data, we used multiple imputation and missing indicator methods. We imputed continuous variables (total serum cholesterol to HDL ratio, systolic blood pressure and body mass index (BMI)) after log transformation. Smoking status and IMD deciles we imputed as ordinal variables. We used five imputations for model one, which had only one imputed clinical variable (smoking), and ten imputations for model two, which included all five imputed variables. We used multiple imputation chained equations (MICE), after ten burn-in iterations, with Stata command mi impute.^26^ We used ordinal logistic regression models for ordinal variables and linear regression for log transformed continuous variables. We assessed imputations for consistency by examining summary statistics and density plots for imputed data and the original dataset.

We also used missing indicator methods for recent blood tests. These were platelet count and C reactive protein. For these we used categorical variables, with missing indicators for unknown values. We categorised CRP results using thresholds used in clinical trials of point-of-care CRP testing: unknown, <5 mg/L, 5–19 mg/L, and 20 mg/L or more.^27^ We categorised platelet results by reference range into: unknown, thrombocytopaenia (<150 × 10^9^/L), within reference range 150 to (450 × 10^9^/L), and thrombocythaemia (>450 × 10^9^/L).

#### Model development

For both regression models, we excluded variables with odds ratios close to one in the initial modelling, and combined some variables, before fitting final models (Supplementary methods).

We used logistic regression, with fractional polynomials to model continuous variables, and did not use stepwise variable selection. We estimated apparent calibration and discrimination.

To choose a statistical model to convert to a clinical points score we considered diagnostic performance, and parsimony. This meant choosing the simpler model unless the more complex model gave a clinically important improvement in performance. To derive the score we specified a scaling factor based on age, so that two points in the score are equivalent to the risk from being 20 years older. We categorised age into 20 year periods, and used the midpoints to calculate risk. We then transformed the risk from other variables into points on this scale, rounding to the nearest point.^28^ We calculated predicted probabilities for the full range of possible scores. For context we calculated the ten-year risk that would achieve the equivalent 28 day risk each month.

#### External validation

We applied the models and score to the external dataset to estimate predicted probabilities. We used these predictions to estimate C statistics, expected/observed ratios, and draw calibration plots. We calculated measures in each imputed dataset and combined them with Rubin’s rules where appropriate. We report median predicted probabilities and expected to observed ratios over the imputed datasets.

Deriving C statistics had high time complexity (the computational time required increased non-linearly with the number of patients). To allow calculation of C statistics in these large datasets we divided each imputed dataset into 20 random subsets and derived the C statistic in each. We then used random effects meta-analysis to combine these results to get the overall C statistic for each imputed dataset (We did this as the default was random effects–there was no heterogeneity so the results would be identical with fixed effects models).

We examined the clinical utility of the statistical models and score using net benefit analysis across the range of predicted probabilities.^29^ Net benefit is calculated by subtracting scaled false positives from true positives, at each predicted probability. The scaling puts the false and true positives on the same scale. It is determined by the level of risk aversion. This is the amount of treatment that would be acceptable in false positives for each necessary treatment, so varies according to the treatment being considered. Different diagnostic strategies can be compared by plotting their net benefit against threshold probability. Net benefit analysis includes plotting the default strategies of treating everyone or no one, as well as comparing decision tools to each other.

#### Model performance at thresholds

We also calculated sensitivity, specificity, positive and negative likelihood ratios, and predictive values at probability and points thresholds. We applied probability thresholds as if they were to be used for a clinical decision, and calculated numbers of patients with true and false positives and negatives, above the threshold per 100,000 peopcaveatle.

To aid comparison within and between each of the logistic regression prediction models and the score, we calculated predictive performance in the external calibration population. As previous studies have concentrated on people with pneumonia as a high-risk group, we also evaluated the performance of this single covariate as a predictor of acute CVD events (Supplementary Table S4).^30^

We also calculated measures of diagnostic performance (sensitivity, specificity, negative and positive likelihood ratios and predictive values) at each point score for the DASHI (Supplementary Table S5).

We used Stata 17 and 18 for statistical analyses (Stata Corp. College Station Tx).

#### Patient and public involvement

This study was part of a larger project on infection-related CVD identification and prevention. We developed the project protocol with the input from PPI participants, who had been patients or carers for patients with experience of acute respiratory infection, cardiac disease, or both. They commented on the proposed work, and suggested changes that we incorporated in the final protocol.

#### Data source and linkages

The cohorts were derived from data in the Clinical Practice Research Datalink (CPRD) GOLD and Aurum databases. These are databases of UK primary care records from different electronic clinical records systems.

Aurum comes from practices in England that used EMIS® software (Egton Medical Information Systems, Leeds, UK), it covers about 13% of the population of England.^31^ GOLD data comes from different practices across the United Kingdom that used Vision® software (Cegedim Healthcare Solutions, London, UK).^32^ Gold covers about 7% of the population.^32^ The model development dataset came from CPRD Aurum. We used CPRD GOLD data for validation. We excluded patients from Aurum if they appeared in both datasets. Both datasets are representative of the wider patient population in terms of deprivation, ethnicity and age.^31^^,^^32^ They provide coded data, rather than free text. Clinicians code the data in the process of routine clinical care, so we are not able to identify the processes that lead to diagnoses.

We used data from 1st January 1999 to 31st December 2019. CPRD datasets are linked to Hospital Episode Statistics (HES) and Office of National Statistics (ONS) data from the start of 1999. The end of the period was the latest available data at the start of the project and preceded the start of the UK’s COVID pandemic. The ONS datasets were Index of Multiple Deprivation (IMD) and ONS mortality data from death certificates.

#### Data linkages

The Clinical Practice Research Datalink (CPRD) provided the primary care data (CPRD GOLD and Aurum from which we extracted the cohorts), to the Nuffield Department of Primary Care Sciences under a departmental licence. CPRD provided data linkages to the study population after extraction. These are person-level data, linked at that level.

#### Index of multiple deprivation

IMD is the official measure of relative deprivation in small areas in England.^33^ The areas have about 1500 people in them. ONS estimates deprivation using a measure that covers seven domains: income, employment, education, skills and training, health and disability, crime, barriers to housing and services, and living environment. They then rank areas by score; IMD is a relative, rather than absolute measure.

Aurum data were linked to IMD data except for 4% which were missing (154,961). GOLD data were linked to IMD data except for 50% which were missing (1,310,635). The discrepancy between these is mostly geographical—small area ONS data is applicable to England only, so patients living in Wales or Scotland are not linkable. Aurum data comes only from England, Gold data are from the whole of the U.K.

#### Mortality

All patients who died in the UK before the data cut was extracted would be included in ONS mortality data. In Aurum there were 110,691 patients who had died after the index date, mostly after the end of the study. Of these there were 1441 CVD deaths in the study period of 28 days post index date. In GOLD 48,689 patients died, and were linked to ONS mortality data. There were 252 CVD deaths in the study follow-up period in the GOLD cohort.

#### Hospital Episode Statistics

All hospital episodes of care are linked to CPRD. Initially patients were identified in CPRD data as having had no prior CVD, being over 40 years and presenting with an acute respiratory tract infection. Linkage to Hospital Episode Statistics data then allowed us to identify more patients with prior CVD, who we excluded (Supplementary Fig. S1).

### Role of funding source

The funders had no role in study design, data collection, data analyses, interpretation, or writing of the report.

## Results

### Study population characteristics

The derivation dataset comprised 3,789,293 patients with first acute respiratory infections (Table 1). The mean age was 56.5 years (standard deviation, SD, 13.7). There were 11,996 cardiovascular events in the following 28 days (0.3%). The validation dataset had 2,636,981 patients. The mean age was 56.7 years (SD 13.6) and 6868 (0.3%) had outcome events. The majority of the populations were female (58%) but females made up 50% of those who had CVD events.

**Table 1:** Characteristics of patients by CVD event outcome status in derivation and validation datasets.

Continuous	Derivation dataset						Validation dataset					
	Total		No CVD		CVD		Total		No CVD		CVD	
	Mean	SD	Mean	SD	Mean	SD	Mean	SD	Mean	SD	Mean	SD
Age in years	56.5	13.7	56.4	13.6	75.0	13.7	56.7	13.6	56.6	13.5	74.7	13.4
Cholesterol: HDL ratio	3.9	1.3	3.9	1.3	3.9	1.3	3.6	1.7	3.6	1.7	3.2	0.8
Systolic BP mmHg	131.2	17.1	131.2	17.1	138.0	20.2	132.0	17.5	132.0	17.5	140.7	20.3
BMI KgM^−2^	27.8	5.8	27.8	5.8	27.0	6.2	27.7	5.7	27.7	5.7	27.0	5.9

Categorical	n	%	n	%	n	%	n	%	n	%	n	%
Total	3,789,293	100%	3,777,297	99.7%	11,996	0.3%	2,636,981	100%	2,630,113	99.7%	6868	0.3%
Female	2,185,255	57.7%	2,179,316	57.5%	5939	49.5%	1,530,454	58.0%	1,527,014	57.9%	3440	50.1%
URTI	2,398,312	63.3%	2,395,640	63.2%	2672	22.3%	1,669,855	63.3%	1,668,200	63.3%	1655	24.1%
LRTI	1,312,569	34.6%	1,305,943	34.5%	6626	55.2%	918,981	34.8%	915,408	34.7%	3573	52.0%
Additional pneumonia diagnosis	78,412	2.1%	75,714	2.0%	2698	22.5%	48,145	1.8%	46,505	1.8%	1640	23.9%
Influenza	189,567	5.0%	188,851	5.0%	716	6.0%	138,216	5.2%	137,818	5.2%	398	5.8%
Smoking status:												
Never smoked	1,359,179	35.9%	1,355,722	35.8%	3457	28.8%	875,671	33.2%	873,908	33.1%	1763	25.7%
Ex-smoker	860,961	22.7%	857,629	22.6%	3332	27.8%	426,837	16.2%	425,609	16.1%	1228	17.9%
Light smoker (<10/day)	131,370	3.5%	130,899	3.5%	471	3.9%	118,481	4.5%	118,175	4.5%	306	4.5%
Moderate smoker (10–19/day)	170,701	4.5%	170,211	4.5%	490	4.1%	184,054	7.0%	183,645	7.0%	409	6.0%
Heavy smoker (20+/day)	122,481	3.2%	122,102	3.2%	379	3.2%	155,509	5.9%	155,108	5.9%	401	5.8%
Smoker, amount unknown	285,596	7.5%	284,684	7.5%	912	7.6%	160,415	6.1%	159,899	6.1%	516	7.5%
Smoking data missing	859,005	22.7%	856,050	22.6%	2955	24.6%	716,014	27.2%	713,769	27.1%	2245	32.7%
Diabetes	300,963	7.9%	298,905	7.9%	2058	17.2%	161,397	6.1%	160,414	6.1%	983	14.3%
Heart failure	36,325	1.0%	35,324	0.9%	1001	8.3%	27,973	1.1%	27,408	1.0%	565	8.2%
Chronic heart disease	49,348	1.3%	48,793	1.3%	555	4.6%	30,684	1.2%	30,438	1.2%	246	3.6%
Atrial arrhythmia	78,807	2.1%	77,480	2.0%	1327	11.1%	50,838	1.9%	50,181	1.9%	657	9.6%
Markers of arterial disease	289,681	7.6%	287,102	7.6%	2579	21.5%	174,204	6.6%	173,022	6.6%	1182	17.2%
Cancer	123,686	3.3%	122,875	3.2%	811	6.8%	70,839	2.7%	70,470	2.7%	369	5.4%
Family history of CVD	26,370	0.7%	26,298	0.7%	72	0.6%	4339	0.2%	4330	0.2%	9	0.1%
Anticoagulants	65,776	1.7%	64,900	1.7%	876	7.3%	42,357	1.6%	41,979	1.6%	378	5.5%
Antiplatelets	15,572	0.4%	15,198	0.4%	374	3.1%	10,815	0.4%	10,623	0.4%	192	2.8%
Antihypertensives	747,893	19.7%	742,457	19.6%	5436	45.3%	498,441	18.9%	495,540	18.8%	2901	42.2%
Rheumatoid arthritis	48,098	1.3%	47,779	1.3%	319	2.7%	34,223	1.3%	34,040	1.3%	183	2.7%
Statins	424,842	11.2%	422,107	11.1%	2735	22.8%	244,312	9.3%	243,156	9.2%	1156	16.8%
Platelets x 10^9^/L												
Unknown platelets	2,654,098	70%	2,647,469	69.9%	6629	55.3%	2,023,120	76.7%	2,018,449	76.5%	4671	68%
Thrombocytopaenia (<150)	34,487	0.9%	34,176	0.9%	311	2.6%	18,250	0.7%	18,108	0.7%	142	2.1%
Platelets normal (150–450)	1,078,154	28.5%	1,073,325	28.3%	4829	40.3%	583,543	22.1%	581,591	22.1%	1952	28.4%
Thrombocytosis (>450)	22,554	0.6%	22,327	0.6%	227	1.9%	12,068	0.5%	11,965	0.5%	103	1.5%
CRP mg/L												
CRP unknown	3,510,766	92.6%	3,500,121	92.4%	10,645	88.7%	2,481,348	94.1%	2,475,011	93.9%	6337	92.3%
CRP < 5	139,785	3.7%	139,380	3.7%	405	3.4%	76,949	2.9%	76,818	2.9%	131	1.9%
CRP 5 to <20	109,479	2.9%	108,943	2.9%	536	4.5%	62,726	2.4%	62,485	2.4%	241	3.5%
CRP ≥ 20	29,263	0.8%	28,853	0.8%	410	3.4%	15,958	0.6%	15,799	0.6%	159	2.3%
Index of multiple deprivation:												
Most deprived decile	336,655	8.9%	335,215	8.8%	1440	12%	92,357	3.5%	91,971	3.5%	386	5.6%
Deprivation missing	97,941	2.6%	97,810	2.6%	131	1.1%	1,310,635	49.7%	1,307,710	49.6%	2925	42.6%

CVD = people with a cardiovascular event outcome in the 28 days following respiratory infection. Respiratory Tract Infection (RTI) categorised into upper (URTI), lower (LRTI) and pneumonia. Influenza is a separate, non exclusive category and can be included separately in addition to LRTI (default for influenza, unless coded to another site). Heart failure includes all non-ischaemic diagnoses. Chronic heart disease incluses valvular disease, hypertensive disease and congenital disease. Atrial arrhythmias include atrial tachicardias, fibrillation, and flutter. Diabetes includes type i, type ii, and other/unrecorded type. Markers of arterial disease includes markers of possible atherosclerosis: chronic kidney disease, peripheral arterial disease, and erectile dysfunction. Cholesterol: HDL ratio is serum total cholesterol/serum HDL cholesterol. CRP id C Reactive Protein. BMI is Body Mass Index.

### Model development

The regression models comprised the clinical variables detailed in Table 2 (for model equations see Supplementary materials). The variable with the strongest association with CVD in models one and two was pneumonia (Odds ratio 10.59, 95% CI 9.98–11.24, in model one and 9.82, 95% CI 9.21–10.46 in model two). Other variables strongly associated with CVD were lower respiratory tract infection (OR 2.59, 95% CI 2.48–2.71, and 2.50 95% CI 2.38–2.63, respectively) and age (OR for each year increase 1.07, 95% CI 1.07–1.07, and 1.06 95% CI 1.06–1.07 respectively). Some variables reduced the predicted probabilities: influenza diagnosis lowered predicted risk in both models (OR 0.84, 95% CI 0.78–0.91, and 0.85, 95% CI 0.78–0.92, respectively). In model two, CRP less than 5 mg/L reduced predicted risk, compared to the baseline of unknown CRP (0.86 (0.77–0.96)). In model two predicted risk was also increased by antiplatelets (OR 2.12 95% CI 1.03–2.38) and anticoagulants (OR 1.13 95% CI 1.03–1.24). Internal apparent performance and calibration results were similar between models (median C statistics 0.88, with good calibration for both models) (Supplementary results: Supplementary Fig. S2 and Table S1).

**Table 2:** Variables included in models, and contributions to risk prediction.

Variables	Model		
Continuous	Model one OR (95% CI)	Model two OR (95% CI)	DASHI score
Age^a^	1.07 (1.07–1.07)	1.06 (1.06–1.07)	40–59 years = 0 points, 60–79 = 2 points, 80+ = 4 points
Cholesterol: HDL ratio		1.10 (1.08–1.13)	
Systolic blood pressure^b^			
Term one		3.67 × 10^−6^ (3.99 × 10^−8^–3.33 × 10^−4^)	
Term two		406.59 (58.56–2822.97)	
BMI^b^			
Term one		0.12 (0.07–0.12)	
Term two		1.08 (1.06–1.11)	
Categorical or binary			
RTI diagnosis			
URTI	Baseline	Baseline	0 points
LRTI	2.59 (2.48–2.71)	2.50 (2.38–2.63)	1 point
Pneumonia	10.59 (9.98–11.24)	9.82 (9.21–10.46)	4 points
Additional influenza diagnosis	0.84 (0.78–0.91)	0.85 (0.78–0.92)	
Smoking status:			
Never smoked	Baseline	Baseline	
Ex-smoker	1.24 (1.19–1.30)	1.14 (1.08–1.20)	0 points for never or ex-smokers
Light smoker (<10/day)	1.69 (1.54–1.87)	1.48 (1.32–1.66)	1 point for any current smoking
Moderate smoker (10–19/day)	1.70 (1.52–1.89)	1.50 (1.36–1.65)	
Heavy smoker (20+/day)	1.90 (1.70–2.12)	1.60 (1.44–1.79)	
Smoker, amount unknown	1.61 (1.50–1.74)	1.44 (1.33–1.56)	
Diabetes	1.49 (1.42–1.57)	1.32 (1.25–1.40)	1 point
Heart failure	1.92 (1.79–2.05)	1.67 (1.55–1.81)	1 point
Markers of arterial disease	1.21 (1.15–1.26)	1.04 (0.99–1.10)	
Male sex		1.43 (1.37–1.50)	
Chronic heart disease		1.30 (1.18–1.43)	
Atrial arrhythmia		1.19 (1.10–1.29)	
Cancer		0.93 (0.86–1.00)	
Family history of CVD		1.28 (1.00–1.65)	
Anticoagulants		1.13 (1.03–1.24)	
Antiplatelets		2.12 (1.03–2.38)	
Antihypertensives		1.30 (1.24–1.36)	
Rheumatoid arthritis		1.20 (1.06–1.36)	
Statins		1.15 (1.09–1.22)	
Platelets x 10^9^/L			
Unknown platelets		Baseline	
Thrombocytopaenia (<150)		1.23 (1.08–1.39)	
Platelets normal (150–450)		1.10 (1.06–1.16)	
Thrombocytosis (>450)		1.32 (1.14–1.53)	
CRP mg/L			
CRP unknown		Baseline	
CRP < 5		0.86 (0.77–0.96)	
CRP 5 to <20		1.10 (1.06–1.16)	
CRP ≥ 20		1.32 (1.14–1.53)	
Index of multiple deprivation^c^			
Least derived decile		Baseline	
Most deprived decile		1.52 (1.40–1.66)	

Respiratory Tract Infection (RTI) categorised into upper (URTI), lower (LRTI) and pneumonia. Influenza is a separate, non exclusive category and can be included separately in addition to LRTI (default for influenza, unless coded to another site). Heart failure includes all non-ischaemic diagnoses. Chronic heart disease incluses valvular disease, hypertensive disease and congenital disease. Atrial arrhythmias include atrial tachicardias, fibrillation, and flutter. Diabetes includes type i, type ii, and other/unrecorded type. Markers of arterial disease includes markers of possible atherosclerosis: chronic kidney disease, peripheral arterial disease, and erectile dysfunction. Cholesterol: HDL ratio is serum total cholesterol/serum HDL cholesterol. CRP is C Reactive Protein.

a Age in models = age in years −56.5. DASHI age points: 40–59 years = 0 points, 60–79 = 2 points, 80+ = 4 points.

b Variable transformed with fractional polynomials, so not directly interpretable. BMI (Body Mass Index) term one is (BMI in KgM^−2^/10)ˆ0.5−1.66, BMI term two is (BMI in KgM^−2^/10)ˆ2−7.61, Systolic BP term one is (systolic BP in mmHg/100)ˆ0.5–1.14, systolic term two is (systolic BP/100)ˆ0.5∗ln (systolic BP/100)−0.31.

c Model for each decile presented in Supplementary materials.

### Prediction model score: DASHI

Due to the similarities in performance of each model, we derived the DASHI score from the simpler model one (Table 2). DASHI is an acronym of the five variables that confer points: Diabetes (1 point for diabetes mellitus of any type), Age (2 points for age 60–79, 4 points for 80+ years), Smoking (1 point for current smokers), Heart failure (1 point for a diagnosis), and Infection type (1 point for LRTI, 4 points for pneumonia). The variables influenza and subcategories of smoking dropped out in the derivation process because they conferred less than half of one point to the score.

The DASHI score predicts 28-day risks from 0.04% for zero points, to 35.6% for the maximum of 11 points (Supplementary Table S2). For context, this table also gives an illustrative ten-year risk that could give patients an equivalent 28-day risk.

### Model performance

Each model demonstrated excellent discrimination with median C statistics of 0.86 (IQR 0.860–0.860) for model one, 0.85 (IQR 0.849–0.852) for model two, and 0.85 (IQR 0.848–0.849) for the DASHI score (Table 3).

**Table 3:** External validation results.

Model	Concordance statistic median (IQR)	Observed to Expected ratio median (IQR)
Model one	0.86 (0.8599–0.8603)	0.83 (0.83–0.83)
Model two	0.85 (0.849–0.852)	0.78 (0.77–0.78)
DASHI score	0.85 (0.848–0.849)	0.85 (0.85–0.85)

**Fig. 1:**
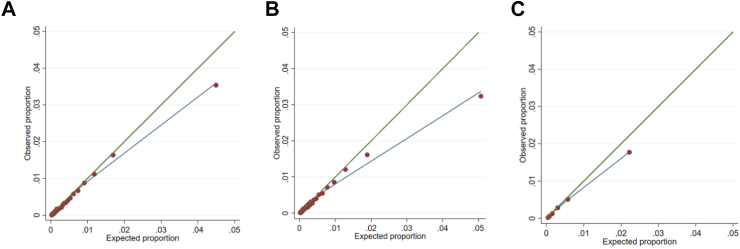
External calibration plots: proportions expected and observed in groups of predicted risk (red markers). Green line—expected equals observed. Blue line—Cubic spline based on red markers. A) Model one, groups are 50ths of expected risk. B) Model two, groups are 50ths of expected risk C) DASHI score, groups are deciles of expected risk.

External calibration was also good. The median observed to expected ratio was 0.83 (IQR 0.83–0.83) for model one, 0.78 (IQR 0.77–0.78) for model two and 0.85 (IQR 0.85–0.85) for the DASHI score (Table 3). There was over prediction of risk of small magnitude at the highest predicted probabilities in all the models (O/E ratio for model one: 0.83 (IQR 0.83–0.83); model two: 0.78 (IQR 0.77–0.78); DASHI: 0.85 (IQR 0.85–0.85) Fig. 1, Table 3). The greatest magnitude overprediction was in model two. According to model two the expected proportion of events in the 2% of the population with the highest risk was ∼5%, but a little over 3% had outcomes.

**Fig. 2:**
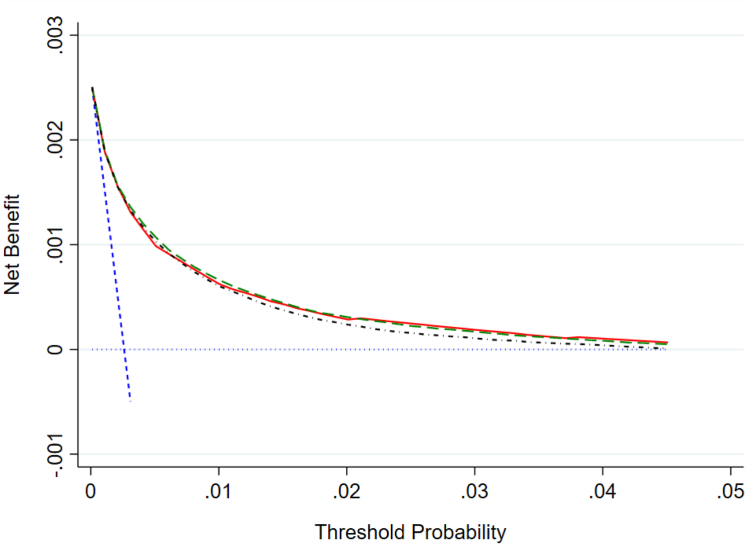
Decision curves for models one, two, and the DASHI score. Lines are net benefit for different strategies: blue dash: ‘treat none’, bold blue dash: ‘treat all’, solid red: DASHI score, green dash: model one, black alternating dots and dashes: model two. Net benefit = true positive proportion minus false positive proportion multiplied by threshold probability/1-threshold probability.

Decision curve analysis showed little difference in net benefit between the two models and DASHI score. They all outperformed the default options of assuming all or none of the patients would have CVD events (Fig. 2).

## Discussion

We derived and validated the first prediction models and risk score for post-acute respiratory infection CVD event risk in a population of patients aged 40 years or older, using data from 6.4 million patients. The DASHI score incorporates five clinical variables: Diabetes, Age, Smoking, Heart failure, and Infection type. It showed excellent discrimination and good calibration upon external validation.

This score could be used in primary care to estimate cardiovascular risk in patients with acute respiratory infection to target adequate primary prevention.

We present a novel tool for predicting post-infection CVD events.^16^ Prior studies found variables associated with infection-related CVD, but these studies were not seeking to predict risk, and clinicians should not be tempted to use these associations to guide treatment decisions.

Causal relationships do not necessarily operate as predictors in the way one might expect. Antiplatelets and anticoagulants reduce CVD events.^34^ Clinicians should not be reassured if their patient is taking these medications–in model two these variables increase the predicted risk, because they are associated with higher underlying risk.

Pneumonia is an important variable in our models and is known to be associated with CVD events.^30^^,^^35^^,^^36^ An observational study examining the effect of aspirin on CVD events in pneumonia suggests a protective effect.^30^ However, a large proportion of post-infection CVD events occur in people with other diagnoses (78% and 76% of events in our derivation and validation data).

When studies have used prediction-modelling methods to estimate CVD risk, they have not targeted post-infection CVD, nor included infection-related variables.^16^ NICE guidance has not advised acute, infection-related risk prediction. Instead, they advise GPs in the UK to estimate overall ten-year CVD risk using the QRisk prediction models.^37^ These models were developed and validated in UK primary care datasets, with comparable methods to this study.

DASHI is the only CVD risk prediction tool that considers respiratory infections, it is simpler to use, and predicts shorter-term risk than most rules.

There are limitations in the data. Clinicians collected these data for a different purpose, over 20 years, during which there were changes in coding, patient behaviour, and clinical practice. However, these inconsistencies apply in both the derivation and validation datasets, and reflect the data that is available in primary care. We had to combine some categories of exposure, and impute some data. We have missed acute respiratory infections that did not present to primary care, or were not coded. This would reduce the size of the dataset, which has remained sufficiently large, but it means the results are only generalizable to presentations recorded in primary care, which is the population for which it is intended.

This study predates the onset of the COVID-19 pandemic in its conception, funding, and the data available at the time of analysis. A further analysis could alter the results because Covid has an association with CVD.^3^ On the other hand, so do many viral and bacterial infections.^4^^,^^8^ A simple explanation is CVD is triggered by the common inflammatory response or physiological disturbances rather than many different effects specific to many different organisms. Non-sterilizing influenza vaccines and Covid vaccines reduce the risk of CVD events, which also points to severity of the illness being important.^38^^,^^39^ As with the other variables, causation does not necessarily increase predicted risk–influenza is known to increase the risk of CVD but in our modelling, influenza first reduced the predicted risk in the regression models, then dropped out of the DASHI score completely, as the effect was too small to confer half of one point and was rounded to zero.

It is possible that DASHI would have performed differently in the first waves of the COVID-19 pandemic, as it might with any pandemic. The risks of CVD following Covid infection were not stable over time—we would expect the CVD risk to be lower now than in the early pandemic because the risk is reduced by covid vaccination, and there is evidence that CVD was underdiagnosed during the early pandemic.^38^, ^39^, ^40^ However, if the differences in CVD risk are driven by reductions in covid causing lower respiratory tract infections and pneumonia, which seems likely, DASHI may still perform well. We used diagnoses recorded in clinical records. We cannot tell how pneumonia was diagnosed, for example, but it is likely that some cases have been diagnosed following chest radiography. Patients with more severe infection are more likely to be assessed in hospital or ambulatory care settings, which may not be coded in the primary care record until discharge. They would then enter the cohort later than those with URTIs. It is also possible that patients with severe infections present sooner, compared to people with minor illnesses. Either way, the presentation to primary care is representative of the population in which the score should be used.

We selected variables with physicians’ expert opinion. This is necessarily a biased process, as clinicians bring their own subjective views and experiences. A strength is that it resulted in a score that is easily calculated, and has face validity with the target users, clinicians.

The calibration of the models and score is imperfect. It is worse at the higher ends of the predicted probabilities. For example, in the top 2% of the population by risk, model one over-predicts by less than one percent. This is small, applies to relatively few patients, and, as they would be high-risk anyway, is unlikely to make much difference in clinical use. The C statistic is of less utility for low prevalence outcomes, and so we have provided decision curve analysis, other diagnostic accuracy measures, and ratios of true and false results to aid interpretation.^41^

Deriving points scores necessarily loses information; they are in effect new models, and need separate validation, which we have done. This resulted in a simpler tool, and the validation shows it performs well. Scores are also easier to implement in clinical practice than statistical models.

Primary care clinicians can use DASHI to estimate and discuss primary CVD event risk. DASHI is valid for patients over the age of 40 presenting to primary care with an acute respiratory tract infection. Many high-risk patients will already be eligible for primary prevention. Clinicians should take the opportunity to offer routine primary prevention with statins to those who are eligible, and to check adherence and optimise the dose in people already prescribed them. As the risks from statins are very low, a low threshold would be appropriate to trigger this action; an equivalent to the 10% ten-year CVD risk used for statin prescriptions is one DASHI point.^37^^,^^42^

DASHI could be re-validated in post-COVID-19 pandemic populations, but researchers should consider the complexities of investigating the early waves and consider using data from after widespread vaccination. A clinician considering additional higher risk interventions, such as short prescription of antiplatelets, could apply a higher threshold. The predicted risk at three DASHI points exceeds the risk of major bleeding from short duration single antiplatelet therapy (about 0.3%).^15^ The CVD event risk at four DASHI points exceeds the risk of major bleeding with dual antiplatelet therapy (0.5%).^15^

Clinicians could also use DASHI to encourage vaccination to prevent cardiovascular events, though the vaccination itself would not be administered during acute illness.^9^^,^^12^ Meta-analysis of previous trials of antibiotics in stable coronary disease shows at best no benefit, and at worst increased mortality.^43^ Acute respiratory infection can be an indication for antibiotic prescribing, but clinicians should not prescribe antibiotics for CVD risk.

DASHI is a simple, validated score for predicting risk of primary CVD events in acute respiratory infections. It applies to primary care populations over the age of 40, presenting with respiratory infections.

This new tool is a way to identify individuals who are most likely to have an infection-related cardiovascular event. It enables research aiming to reduce CVD events in people with acute respiratory infections and may encourage uptake of primary prevention measures. An intervention trial of intensified cardiovascular prevention in patients with high DASHI score in the weeks following infection is warranted.

## Contributors

Authors contributed to the following:

Study design and planning: JJL, CK, CWD, JAD, CWG, FDRH, JPS.

Conduct/analyses of the study, verification of datasets: JJL, CK, CWD.

Reporting, editing and writing the paper: JJL, CK, CWD, JAD, CWG, FDRH, JPS.

Guarantor: JPS.

All authors read and approved the final version of the manuscript. JJL and CWD have verified the underlying data. The corresponding author attests that all listed authors meet authorship criteria and that no others meeting the criteria have been omitted.

## Data sharing statement

We are unable to share data, which is available from CPRD. Software is available commercially from StataCorp.

## Declaration of interests

JJL and this study were funded by doctoral fellowship funding from the NIHR [NIHR300738]. JJL is partly supported by NIHR ARC OTV.

CK and JS declare support from the Wellcome Trust/Royal Society, NIHR School for Primary Care and NIHR Oxford Biomedical Research Centre.

James P. Sheppard receives funding from the Wellcome Trust/Royal Society via a Sir Henry Dale Fellowship (ref: 211182/Z/18/Z), the National Institute for Health and Care Research (NIHR) and from the British Heart Foundation (refs: PG/21/10341; FS/19/13/34235). JPS additionally declares payments from DoctorLink for critical reviews of their evidence synthesis exercises and payments for participation in the Data Safety Monitoring Board of the Hypertension Treatment in Nigeria Study sponsored by Northwestern University, US. He has an unpaid role as Secretary and Trustee of the British and Irish Hypertension Society.

CWD and JD declare no support for the present project. FRDH is chair of EPCCS and IPCCS. He declares occasional fees/expenses for advisory work or speaking for AZ, BI, BMS/Pfizer.

CWG is supported by a Wellcome Career Development Award (225868/Z/22/Z).

## Footnotes

d In order of expert ranking: Age, heart failure, diabetes, smoking status, renal disease, hypertension, peripheral vascular disease, COPD, systolic and diastolic BP, sex, total cholesterol to HDL cholesterol ratio, BMI, Atrial arrhythmia, dementia, anticoagulation, non-steroidal anti-inflammatory prescriptions, obstructive sleep apnoea, antiplatelet use, antihypertensives, statins, rheumatoid arthritis, platelets, CRP, erectile dysfunction, learning disability, chronic or congenital heart disease, IMD score, cancers (solid and haematological), family history of CVD, pneumococcal vaccine, glucose lowering medications, chemotherapy in the last 6 months, influenza vaccine, white cell count, haemoglobin, lupus, asthma, antibiotics on the day of consultation, splenic dysfunction, immunosuppression, connective tissue disorders, ethnicity, alcohol consumption (moderate or heavy), other chronic lung disease, chronic liver disease, migraine, coeliac disease, drugs that cause bleeding (from BNF), recent cellulitis, seizure disorders, other chronic neurological conditions, UTI number in last 5 years.

## References

[1] Visseren F.L.J., MacH F., Smulders Y.M. (2021). 2021 ESC Guidelines on cardiovascular disease prevention in clinical practiceDeveloped by the Task Force for cardiovascular disease prevention in clinical practice with representatives of the European Society of Cardiology and 12 medical societies with the special contribution of the European Association of Preventive Cardiology (EAPC). Eur Heart J.

[2] Warren-Gash C., Hayward A.C., Hemingway H. (2012). Influenza infection and risk of acute myocardial infarction in england and wales: a CALIBER self-controlled case series study. J Infect Dis.

[3] Katsoularis I., Fonseca-Rodríguez O., Farrington P. (2021). Risk of acute myocardial infarction and ischaemic stroke following COVID-19 in Sweden: a self-controlled case series and matched cohort study. Lancet.

[4] Warren-Gash C., Smeeth L., Hayward A.C. (2009). Influenza as a trigger for acute myocardial infarction or death from cardiovascular disease: a systematic review. Lancet Infect Dis.

[5] Smeeth L., Thomas S.L., Hall A.J. (2004). Risk of myocardial infarction and stroke after acute infection or vaccination. N Engl J Med.

[6] Kwong J.C., Schwartz K.L., Campitelli M.A. (2018). Acute myocardial infarction after laboratory-confirmed influenza infection. N Engl J Med.

[7] Barnes M., Heywood A.E., Mahimbo A. (2015). Acute myocardial infarction and influenza: a meta-analysis of case-control studies. Heart.

[8] Pitman R.J., Melegaro A., Gelb D. (2007). Assessing the burden of influenza and other respiratory infections in England and Wales. J Infect.

[9] Clar C., Oseni Z., Flowers N. (2015). Influenza vaccines for preventing cardiovascular disease. Cochrane Database Syst Rev.

[10] Udell J.A., Zawi R., Bhatt D.L. (2013). Association between influenza vaccination and cardiovascular outcomes in high-risk patients: a meta-analysis. JAMA.

[11] Davidson J.A., Banerjee A., Smeeth L. (2021). Risk of acute respiratory infection and acute cardiovascular events following acute respiratory infection among adults with increased cardiovascular risk in England between 2008 and 2018 : a retrospective , population-based cohort study. Lancet Digit Health.

[12] Jaiswal V., Ang S.P., Lnu K. (2022). Effect of pneumococcal vaccine on mortality and cardiovascular outcomes: a systematic review and meta-analysis. J Clin Med.

[13] (2023). Suspected acute respiratory infection in over 16s: assessment at first presentation and initial management NICE guideline.

[14] Johnston S.C., Easton J.D., Farrant M. (2018). Clopidogrel and aspirin in acute ischemic stroke and high-risk TIA. N Engl J Med.

[15] Prasad K., Siemieniuk R., Hao Q. (2018). Dual antiplatelet therapy with aspirin and clopidogrel for acute high risk transient ischaemic attack and minor ischaemic stroke: a clinical practice guideline. BMJ (Online).

[16] Damen J.A.A.G., Hooft L., Schuit E. (2016). Prediction models for cardiovascular disease risk in the general population: systematic review. BMJ.

[17] Hippisley-Cox J., Coupland C., Brindle P. (2017). Development and validation of QRISK3 risk prediction algorithms to estimate future risk of cardiovascular disease: prospective cohort study. BMJ.

[18] Lee J.J., Bankhead C., Smith M. (2018). Risk factors for influenza-related complications in children during the 2009/10 pandemic : a UK primary care cohort study using linked routinely collected data. Epidemiol Infect.

[19] Sharma A., Pagidipati N.J., Califf R.M. (2020). Impact of regulatory guidance on evaluating cardiovascular risk of new glucose-lowering therapies to treat type 2 diabetes mellitus: lessons learned and future directions. Circulation.

[20] Davidson J.A., Banerjee A., Muzambi R. (2019). Validity of acute cardiovascular outcome diagnoses in European electronic health records: a systematic review protocol. BMJ Open.

[21] CPRD (2021).

[22] Ebell M.H., Afonso A. (2011). A systematic review of clinical decision rules for the diagnosis of influenza. Ann Fam Med.

[23] Ramsay M. (2023).

[24] Riley R.D., Snell K.I.E., Ensor J. (2019). Minimum sample size for developing a multivariable prediction model: PART II - binary and time-to-event outcomes. Stat Med.

[25] Riley R.D., Snell K.I.E., Archer L. (2024). Evaluation of clinical prediction models (part 3): calculating the sample size required for an external validation study. BMJ.

[26] van Buuren S. (2007). Multiple imputation of discrete and continuous data by fully conditional specification. Stat Methods Med Res.

[27] Verbakel J.Y., Lee J.J., Goyder C. (2019). Impact of point-of-care C reactive protein in ambulatory care : a systematic review and meta-analysis. BMJ Open.

[28] Bonnett L.J., Snell K.I.E., Collins G.S. (2019). Guide to presenting clinical prediction models for use in clinical settings. BMJ.

[29] Vickers A.J., Van Calster B., Steyerberg E.W. (2016). Net benefit approaches to the evaluation of prediction models, molecular markers, and diagnostic tests. BMJ.

[30] Hamilton F., Arnold D., Henley W. (2020). Aspirin reduces cardiovascular events in patients with pneumonia: a prior event rate ratio analysis in a large primary care database. Eur Respir J.

[31] Wolf A., Dedman D., Campbell J. (2019). Data resource profile: clinical practice research Datalink (CPRD) Aurum. Int J Epidemiol.

[32] Herrett E., Gallagher A.M., Bhaskaran K. (2015). Data resource profile: clinical practice research Datalink (CPRD). Int J Epidemiol.

[33] GOV.UK (2019).

[34] Collins R., Peto R., Hennekens C. (2009). Aspirin in the primary and secondary prevention of vascular disease: collaborative meta-analysis of individual participant data from randomised trials. Lancet.

[35] Lange P., Vestbo J., Nyboe J. (1995). Risk factors for death and hospitalization from pneumonia. A prospective study of a general population. Eur Respir J.

[36] Corrales-Medina V.F., Serpa J., Rueda A.M. (2009). Acute bacterial pneumonia is associated with the occurrence of acute coronary syndromes. Medicine.

[37] National Institute for Clinical Excellence (2023). NICE guidance CG238.

[38] Xu Y., Li H., Santosa A. (2024). Cardiovascular events following coronavirus disease 2019 vaccination in adults: a nationwide Swedish study. Eur Heart J.

[39] Ip S., North T.L., Torabi F. (2024). Cohort study of cardiovascular safety of different COVID-19 vaccination doses among 46 million adults in England. Nat Commun.

[40] Banerjee A., Chen S., Pasea L. (2021). Excess deaths in people with cardiovascular diseases during the COVID-19 pandemic. Eur J Prev Cardiol.

[41] Romero-Brufau S., Huddleston J.M., Escobar G.J. (2015). Why the C-statistic is not informative to evaluate early warning scores and what metrics to use. Crit Care.

[42] Cai T., Abel L., Langford O. (2021). Associations between statins and adverse events in primary prevention of cardiovascular disease: systematic review with pairwise, network, and dose-response meta-analyses. BMJ.

[43] Gluud C., Als-Nielsen B., Damgaard M. (2008). Clarithromycin for 2 weeks for stable coronary heart disease: 6-Year follow-up of the CLARICOR randomized trial and updated meta-analysis of antibiotics for coronary heart disease. Cardiology.

