# Supplementary material for: Development and external validation of a risk prediction score (DASHI) for cardiovascular events following acute respiratory infections: derivation and validation retrospective cohort study
Source: eClinicalMedicine. 2025 Jun 2;84:103273. doi: 10.1016/j.eclinm.2025.103273 (PMC12167834; doi:10.1016/j.eclinm.2025.103273)
Supplement: Supplementary Appendix [file mmc1.docx]

# Supplementary Appendix

## Supplementary methods:

Data source and linkages

The cohorts were derived from data in the Clinical Practice Research Datalink (CPRD) GOLD and Aurum databases. These are databases of UK primary care records from different electronic clinical records systems.

Aurum comes from practices in England that used EMIS® software (Egton Medical Information Systems, Leeds, UK), it covers about 13% of the population of England.[1] GOLD data comes from different practices across the United Kingdom that used Vision® software (Cegedim Healthcare Solutions, London, UK).[2] Gold covers about 7% of the population.[2] The model development dataset came from CPRD Aurum. We used CPRD GOLD data for validation. We excluded patients from Aurum if they appeared in both datasets. Both datasets are representative of the wider patient population in terms of deprivation, ethnicity and age.[1,2] They provide coded data, rather than free text. Clinicians code the data in the process of routine clinical care, so we are not able to identify the processes that lead to diagnoses.

We used data from 1^st^ January 1999 to 31^st^ December 2019. CPRD datasets are linked to Hospital Episode Statistics (HES) and Office of National Statistics (ONS) data from the start of 1999. The end of the period was the latest available data at the start of the project and preceded the start of the UK’s COVID pandemic. The ONS datasets were Index of Multiple Deprivation (IMD) and ONS mortality data from death certificates.

### *Data Linkages*

The Clinical Practice Research Datalink (CPRD) provided the primary care data (CPRD GOLD and Aurum from which we extracted the cohorts), to the Nuffield Department of Primary Care Sciences under a departmental licence. CPRD provided data linkages to the study population after extraction. These are person-level data, linked at that level.

#### Index of Multiple deprivation

IMD is the official measure of relative deprivation in small areas in England.[3] The areas have about 1,500 people in them. ONS estimates deprivation using a measure that covers seven domains: income, employment, education, skills and training, health and disability, crime, barriers to housing and services, and living environment. They then rank areas by score; IMD is a relative, rather than absolute measure.

Aurum data were linked to IMD data except for 4% which were missing (154,961). GOLD data were linked to IMD data except for 50% which were missing (1,310,635). The discrepancy between these is mostly geographical – small area ONS data is applicable to England only, so patients living in Wales or Scotland are not linkable. Aurum data comes only from England, Gold data are from the whole of the U.K.

#### Mortality

All patients who died in the UK before the data cut was extracted would be included in ONS mortality data. In Aurum there were 110,691 patients who had died after the index date, mostly after the end of the study. Of these there were 1,441 CVD deaths in the study period of 28 days post index date. In GOLD 48,689 patients died, and were linked to ONS mortality data. There were 252 CVD deaths in the study follow-up period in the GOLD cohort.

#### Hospital Episode Statistics

All hospital episodes of care are linked to CPRD. Initially patients were identified in CPRD data as having had no prior CVD, being over 40 years and presenting with an acute respiratory tract infection. Linkage to Hospital Episode Statistics data then allowed us to identify more patients with prior CVD, who we excluded (Figure S1).

Figure S1: Flow chart of patient numbers and exclusion following linkage to HES

Initial population CPRD GOLD 2,651,418

Final population 2,636,981

Prior CVD identified in HES 14,437 (0.5%)

Initial population CPRD Aurum 3,846,313

Final population 3,789,293

Prior CVD identified in HES 57,020 (1.5%)

### Population

We chose to include people over the age of 40 years because of a low risk of events in people under this age. There is also precedent for using this cutoff – it was used in QRisk2 - the clinical model most used for stratifying the primary prevention population in the UK.[4] We used 28 days as this period has been consistently associated with an increase in risk following infections in primary care.[5]

It is possible that some patients had prior CVD, but this was not recorded. We suspect this would be minimal, as this misclassification would require no record in either primary care or secondary care records of an important event. This would reflect the information available to the GP at use of the model, and so clinical reality.

### Outcome

The outcome is a combination of cardiovascular events, within 28 days of the acute respiratory infection. This was a composite of new diagnoses of myocardial ischaemia, stroke, TIA, or deaths from these. We thought this approach came close to the ‘four point Major Adverse Cardiovascular Event (MACE)’ outcome used in trials of antidiabetic medications and largely caused by atherosclerosis.[6] We could not use ischaemic stroke as an outcome, because stroke type is poorly differentiated in CPRD data.[7] We did not include generic ‘heart failure’ diagnosis in the outcome, as it may be due to aetiology other than ischaemia, though we included specifically coded new ischaemic cardiomyopathy. We did not include peripheral vascular events such as limb ischaemia or claudication in the outcome. Instead, we included markers of peripheral arterial disease in the candidate predictors. Similarly, we did not include other chronic cardiac conditions in the outcome as they would not be triggered by a RTI (valve disease, congenital disease etc) and so included chronic heart disease as a predictor instead.

As we used routinely collected datasets there was no blinding of the outcome assessors – and no risk of this study biasing these clinicians’ historic assessments.

The outcome we derived from CPRD, ONS mortality and HES datasets. Hospital Episode Statistics data includes diagnosis data and procedure data.

CPRD recommend the use of search strategies rather than specific codelists.[8] We searched CPRD code files using Stata. We aimed to make code lists specific, rather than over-sensitive. As an example, we did not use codes for referrals to chest pain or TIA clinics in the outcome, as many of these patients go on have CVD events ruled out.

### Codes employed:

CPRD codes do not map directly to ICD codes, and so we had to develop our own codelists. The type and site of infection is very likely prone to misclassification. In the UK primary care situation diagnosis is clinical, without test. For example, clinical risk scores for influenza have diagnostic performance that is moderately predictive.[9] The imperfect data reflects the information that the clinicians would have and is likely a proxy for severity of infection. Misclassification would bias towards a lack of association between the misclassified variables and CVD, but despite this we have shown a strong association with outcomes, so it does not appear to have greatly impacted the validation results.

The CPRD codes we used can be found here: <https://github.com/Protocols-For-Research/CPRD-codes-CVD-infection-risk>. Here follows the ICD and OPCS codes used for the outcome.

#### ICD codes used to identify CVD deaths in ONS data:

Cerebrovascular: I63 I636 I634 I631 I633 I630 I635 I632 I639 I61 I613 I614 I611 I610 I612 I615 I616 I619 I629 I676 I621 I661 I651 I652 I663 I66 I660 I664 I653 I668 I658 I662 I65 I669 I659 I650 I638 I618 I62 I608 I693 I69 I691 I692 I694 I690 I64 I60 I602 I604 I600 I607 I601 I606 I603 I605 I609

Cardiac: I46 I460 I469 I516 I249 I21 I219 I214 I210 I211 I212 I213 I201 I209 I250 I251 I231 I23 I25 I259 I254 I240 I241 I230 I255 I24 I238 I248 I208 I258 I233 I234 I235 I256 I22 I220 I221 I228 I229 I461 I200 I232

#### NHS ‘opcs’ procedure codes used in HES data to identify CVD events

K424 K412 K454 K431 K442 K458 K499 K402 K758 K429 K432 K434 K498 K483 K451 K754 K45 K40 K503 K43 K459 K455 K456 K433 K493 K414 K46 K41 K404 K759 K501 K453 K438 K42 K409 K491 K403 K423 K44 K468 K408 K509 K494 K751 K421 K452 K449 K75 K419 K418 K422 K469 K428 K401 K49 K413 K753 K411 K492 K508 K50 K441 K439 K482 K448 K504 K752 L372 K471 L318 L303 L314 L31 K502 L311 L319

### Predictor selection

We included infection type in all models. Four General Practitioners (GPs) with special interest in cardiology helped rank the other clinical variables. We asked them to rank the relevance of 54 variables^[[1]](#footnote-1)^ from minus three to three. We asked them to consider the completeness of coding as well as clinical relevance. We standardised each GP’s ratings into Z scores to express the relative importance they gave each variable on the same scale, with a mean of zero and a standard deviation of one. We then combined these scaled scores by arithmetic mean across GPs to give a mean Z score. We used this overall mean ranking to order the variables. Model one included those variables with a mean Z score >1 and model two included variables with a mean Z score >0. Variables with a mean Z score of 0 or less were not included in models.

By ‘clinical variable’, we mean a diagnosis, demographic, or test, as ranked by the experts, rather than a term in a statistical model. For example, smoking status was a single clinical variable for clinicians to rank but is represented in the statistical models as multiple categorical variables. These represent people who have never smoked, ex-smokers, light smokers (<10 cigarettes per day), moderate smokers (10-19 cigarettes per day), heavy smokers (20+ cigarettes per day), and those who smoke an unquantified amount.

We derived all the predictors from the clinical records and linked datasets (we did not collect other data). We extracted covariates from the clinical record before the index date. We had to define a relevant time window before the infection to search for relevant codes. We used different time windows for different variables. For cholesterol to HDL ratio, BMI, and systolic blood pressure, we used the most recent record in the five years before the index date. Cancers also had a five-year limit. We took codes for other diagnoses, and family history, from the entire record prior to the index date. In order to better identify ex-smokers, and the amount people smoked, we used the two most recent smoking records. We defined smoking categories as: never smoked, ex-smoker, light (<10 cigarettes per day), medium (10-19 cigarettes per day), heavy (20+ cigarettes per day) and amount unknown.

### Refinement of variables

#### Respiratory infections

The coding systems shaped the respiratory infection diagnosis variable. We were able to classify some respiratory infections into exclusive groups: upper respiratory tract infection (URTI), lower respiratory tract infection (LRTI), and LRTI with pneumonia diagnosis are categories without overlap – codes suggesting infections from the trachea up we counted as URTI, those below this were LRTIs, and specific codes for pneumonia, or it’s complications, were used for pneumonia. Influenza was not an exclusive category – there are codes for influenza without specifying severity or site, but other codes for influenza associated with URTIs, LRTIs, and pneumonia. Influenza is therefore not an exclusive category. We classed exacerbations of Chronic Obstructive Pulmonary Disease (COPD) as lower respiratory tract infection, unless there were codes indicating the exacerbation was a pneumonia. To avoid collinearity COPD was not included as a separate variable.

#### Covariates

We refined, combined and dropped some of the clinical categories after the clinical experts had prioritized them. These were COPD, hypertension, dementia, diabetes subtypes, non-steroidal anti-inflammatories, erectile dysfunction, chronic kidney disease, peripheral vascular disease, cancer subtypes, family history of CVD, and vaccination status.

The top ranked variables for model one, before refinement into the final model were: Age, heart failure, diabetes, smoking status, chronic kidney disease, peripheral vascular disease and COPD. For initial model two, we included those in model one plus variables further down the ranking. The ranking continued: systolic blood pressure, sex, cholesterol to HDL ratio, BMI, atrial arrhythmias, dementia, anticoagulants, NSAIDS, antiplatelets, antihypertensives, rheumatoid arthritis, statin use, platelets, CRP, erectile dysfunction, other chronic heart diseases (including valve disease, congenital disease), IMD decile, haematological cancers, solid cancers, family history of CVD in first degree relative less than 60 years of age, and Pneumococcal vaccine.

We first defined family history of CVD as an event in a first-degree relative aged less than sixty, reflecting the definition used by Qrisk3.[4] Unfortunately, the coding systems do not include codes for this. Instead, we defined a high-risk family history as being a CVD aged less than 65 years if the first-degree relative was female, and fifty-five if male.

We simplified variables by combining rare and overlapping categories. We combined diabetes mellitus type one (which was rare), with type two diabetes and diabetes of other and unspecified types. We did not include the strongly correlated ‘glucose lowering medications’. Haematological cancers were uncommon, so we combined them with solid cancers. We also combined peripheral vascular disease with chronic kidney disease and erectile dysfunction. These three predictors were sparse, and are diagnoses that can have common cause in underlying atherosclerosis. Over the course of the study, the UK was introducing various pneumococcal vaccines against different serotypes, and for different populations (for over 65’s from 2003 for example), so we did not include pneumococcal vaccination as a predictor.[10] Dementia was the only variable excluded on the basis of odds ratio, which was 1.00. No variable selection methods were used that rely on p values because we considered it likely the size of the dataset would lead to very small p values for every variable.

### C statistic calculation

Deriving C statistics had high time complexity (the computational time required increases non-linearly with the number of patients). To allow calculation of C statistics in these large datasets we divided each imputed dataset into 20 random subsets and derived the C statistic in each. We then used random effects meta-analysis to combine these results to get the overall C statistic for each imputed dataset (We did this as the default was random effects - there was no heterogeneity so the results would be identical with fixed effects models).

### Model performance at thresholds

To aid comparison within and between each of the logistic regression prediction models and the score, we calculated predictive performance in the external calibration population. As previous studies have concentrated on people with pneumonia as a high-risk group, we also evaluated the performance of this single covariate as a predictor of acute CVD events (Table S4).[11]

To do this we applied thresholds, and calculated numbers of patients with true and false positives and negatives, were that threshold used to guide a clinical decision in 100,000 patients (Table S4).

We also calculated measures of diagnostic performance (sensitivity, specificity, negative and positive likelihood ratios and predictive values) at each point score for the DASHI (Table S5).

### Sample size calculation

We calculated sample size calculations for model derivation before study protocol approval using methods by Riley *et al*.[12] We based the calculation on preliminary counts in CPRD Gold data; this gave us a conservative outcome prevalence of 0.089%. We aimed for a global shrinkage factor of >0.995. We assumed a maximum of 50 candidate variables (including transformations and interactions). We also checked we would meet the criteria of an absolute difference of <0.05 in apparent and adjusted Nagelkerke's R2, and a margin of error in outcome proportion estimates for null model <0.05. We calculated 61,198 patients would be enough to achieve these criteria.

We also performed a post-hoc sample size calculation for model validation. Derivation sample sizes tend to need to be larger than validation samples, so we relied on this in the protocol. In addition, at the point of writing the protocol we did not have the results from the derivation to use for the calculation. We used the user written Stata package pmvalsampsize, using estimates from internal calibration of the DASHI score.[13] We specified a prevalence of 0.3%, a C statistic of 0.84, and a calibration slope of 1.07. We modelled the distribution of log predicted probabilities with the results from the development data: a skewed normal distribution with a mean of -7.074499 and a variance of 1.030443, skewness of 1 and kurtosis of 4. This returned a result of 115,597 patients being required, with estimating the calibration slope as the most data hungry calculation (observed to expected ratio required 5,510 and the C statistic 20,525). Our dataset exceeded these numbers.

## Supplementary results:

### Model specification

#### Model one

Variable transformations:

IAge__1 = Age-56.70140054.

Model:

logit (p) = intercept + (IAge__1 * .065120631558634) + (Ex-smoker * .216435085481023) + (Smoking <10 per day * .521069474752047) + (Smoking 11 to <20 per day * .6212762224915964) + (Smoking 20 or more per day * .6402038495993185) + (Smoker unknown amount * .479095173673624) + (Heart failure * .6504444223129626) + (Diabetes * .4020472246812231) + (Peripheral vascular disease/erectile dysfunction/CKD * .1866634297003109) + (Lower respiratory tract infection * .9504990322745389) + (Influenza * -.1766913795845679) + (Pneumonia * 2.360004208451382)

#### Model two

Variable transformations:

IAge__1 = Age-56.4915727

IBMI__1 = X^.5-1.660968746 (where: X = BMI/10)

IBMI__2 = X^2-7.611072204 (where: X = BMI/10)

ISyst__1 = X^.5-1.143992165 (where: X = Systolic blood pressure/100)

ISyst__2 = X^.5*ln(X)-.3077889054 (where: X = Systolic blood pressure /100)

Ichol__1 = choloverHDL-3.928200556

Model:

logit (p) = intercept + (IAge__1 * .0624268656055371) + (Male * .3602814828580722) + (IBMI__1 * -2.11821951601376) + (IBMI__2 * .079928616816893) + (ISyst__1 * -12.51473706712241) + (ISyst__2 * 6.00780703111644) + (Ichol__1 * .0994292795556542) + (Ex-smoker * .1285313176342534) + (Smoking <10 per day * .3903047381201049) + (Smoking 11 to <20 per day * .4053342599263999) + (Smoking 20 or more per day * .4722622955322949) + (Smoker unknown amount * .3665724928985887) + (Heart failure * .5156211258528431) + (Diabetes * .277791542462455) + (Peripheral vascular disease/erectile dysfunction/CKD * .0402272711850079) + (Lower respiratory tract infection * .9163506575774839) + (Influenza * -.1650086994783002) + (Pneumonia * 2.284118276574413) + (Chronic heart disease * .2620935008602651) + (Atrial arrhythmias * .1777328854785878) + (Anticoagulated * .1194084995233393) + (Antihypertensives * .2593188135199826) + (Antiplatelets * .7495876582572326) + (Rheumatoid Arthritis * .1847157658361301) + (Statins * .1440367605302275) + (Family history of CVD * .2495579573416379) + (Cancer * -.0752826901643886) + (IMD_decile2 * .0854324013423883) + (IMD_decile3 * .0499679427695114) + (IMD_decile4 * .0824179242752817) + (IMD_decile5 * .1562734961824674) + (IMD_decile6 * .178860458895174) + (IMD_decile7 * .1919099331725266) + (IMD_decile8 * .3073143511672598) + (IMD_decile9 * .3131420516485625) + (IMD_decile10 * .4202440161178939) + (CRP<5 * -.1564572668463038) + (CRP>=5 & CRP<20 * .0243215508572336) + (CRP >=20 * .2480620256490901) + (Platelets <150 * .2041245574197942) + (Platelets >=150 & <450 * .0998278924632811) + (Platelets >=450 * .2784685782020367)

We presented the equations for the models without the intercepts. This is to retain the intellectual property, to allow potential future implementation by clinical software.

DASHI score:

Table S1: DASHI Points Allocation

| Variable | | Points |
| --- | --- | --- |
| Diabetes | No | 0 |
|  | Yes | 1 |
| Age (years) | 40-59 | 0 |
|  | 60-79 | 2 |
|  | 80+ | 4 |
| Smoker | Never, or ex-smoker | 0 |
|  | Current smoker | 1 |
| Heart failure | No | 0 |
|  | Yes | 1 |
| Infection | Upper tract | 0 |
|  | Lower tract | 1 |
|  | Pneumonia | 4 |

Table S2: DASHI predicted risks

| Points scored | Predicted CVD within 28 days (%) | 10-year risk required to give equivalent monthly risk (%) |
| --- | --- | --- |
| 0 | 0.04 | 5.42 |
| 1 | 0.08 | 10.14 |
| 2 | 0.16 | 18.53 |
| 3 | 0.30 | 32.48 |
| 4 | 0.58 | 52.86 |
| 5 | 1.10 | 76.28 |
| 6 | 2.09 | 93.58 |
| 7 | 3.93 | 99.46 |
| 8 | 7.28 | 99.99 |
| 9 | 13.09 | 99.99 |
| 10 | 22.40 | 99.99 |
| 11 | 35.64 | 99.99 |
| Ten year risk calculated as 130 independent 28 day periods using formula 10 year risk = 1-(1-28 day predicted probability)^130^ | | |

### Apparent calibration

As part of the model development process, we internally validated the models. We did not apply methods to adjust for optimism, such as shrinkage factors, as we planned to calibrate externally.

Figure S2: apparent calibration plots

| 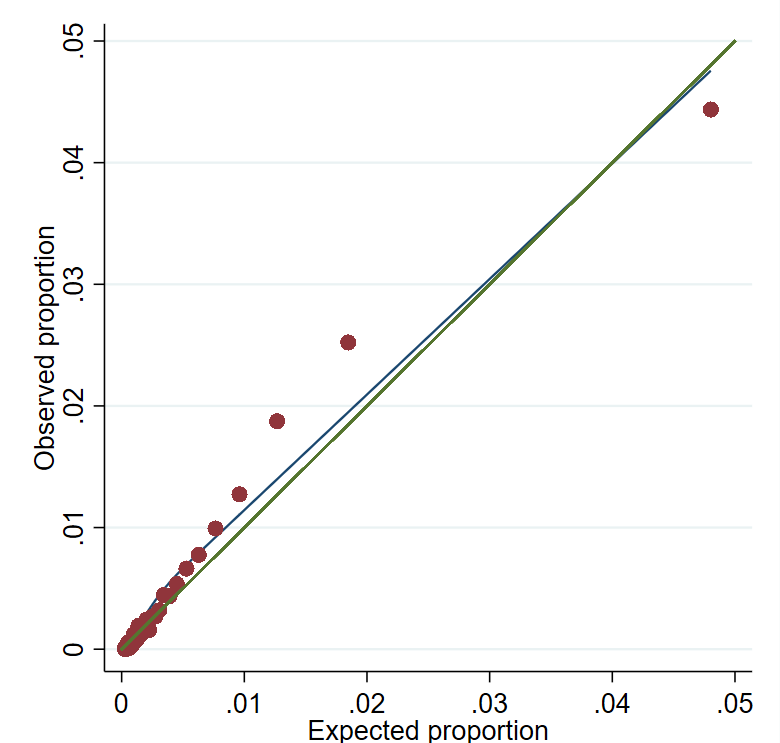 A | 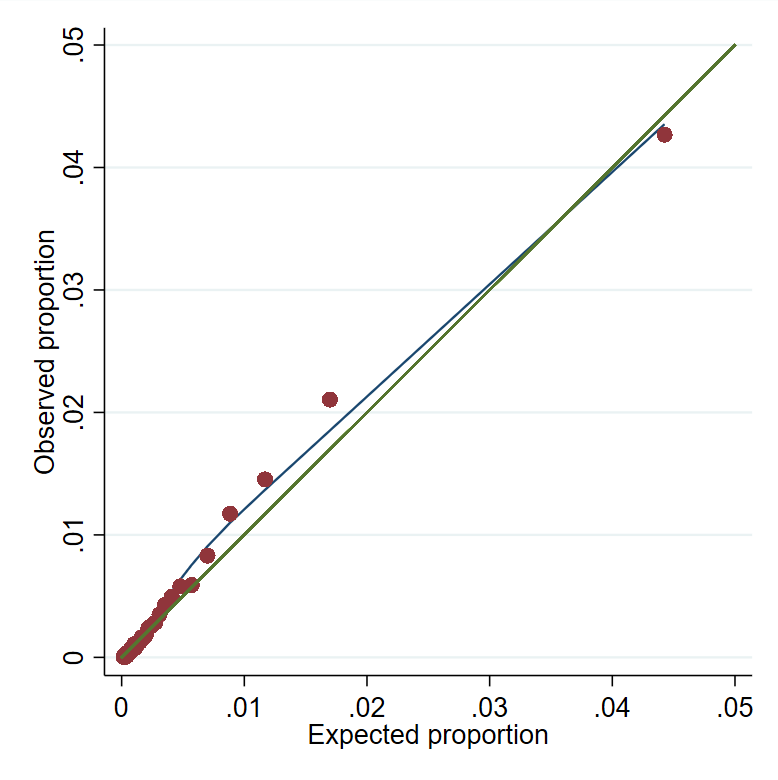  B | 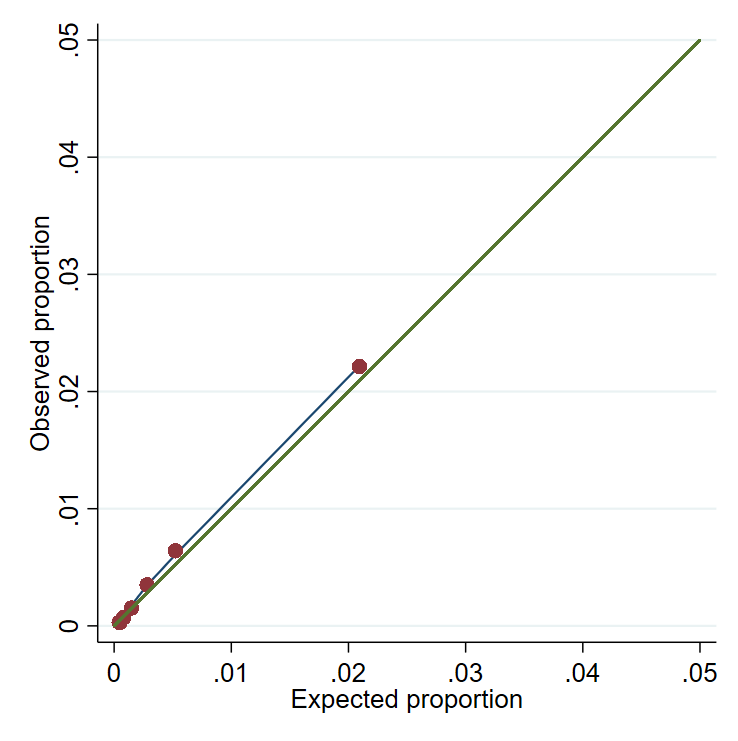 C |
| --- | --- | --- |
| Internal calibration plots: proportions expected and observed in groups of predicted risk (red markers). Green line – expected equals observed. Blue line – Cubic spline based on red markers. A) Model one, groups are 50ths of expected risk B) Model two, groups are 50ths of expected risk C) DASHI score, groups are deciles of expected risk. | | |

### Apparent performance

Table S3: Apparent discrimination and calibration

| Model | C statistic  Median (IQR) | Observed to Expected ratio Median (IQR) |
| --- | --- | --- |
| Model One | 0.88 (0.8782 to 0.8774) | 0.98 (0.9784 to 0.9799) |
| Model Two | 0.88 (0.8842 to 0.8847) | 1.09 (1.0893 to 1.0898) |
| DASHI score | 0.84 (0.8390 to 0.8393) | 1.07 (1.0669 to 1.0668) |

Table S4: External performance: discrimination at thresholds of predicted probability

|  | **Per 100,000 patients** | | | | **Ratios** | |
| --- | --- | --- | --- | --- | --- | --- |
| **Model, predicted probability or points** | **True positives** | **False Positives** | **False Negatives** | **True negatives** | **Ratio of false positives to true positives** | **Ratio of true negatives to false negatives** |
| Prevalence of outcome 260/100K (0.26%) | | | | | | |
| **Model 1** | | | | | | |
| 0.1% | 246 | 51796 | 15 | 47944 | 211 | 3258 |
| 0.2% | 222 | 31222 | 39 | 68518 | 141 | 1768 |
| 1% | 127 | 6023 | 133 | 93717 | 47 | 703 |
| 2% | 75 | 2143 | 186 | 97596 | 29 | 526 |
| 3% | 53 | 1143 | 207 | 98597 | 22 | 474 |
| **Model 2** | | | | | | |
| 0.1% | 241 | 48703 | 19 | 51037 | 202 | 2681 |
| 0.2% | 218 | 30109 | 42 | 69631 | 138 | 1656 |
| 1% | 127 | 6595 | 133 | 93144 | 52 | 700 |
| 2% | 77 | 2608 | 184 | 97132 | 34 | 529 |
| 3% | 53 | 1390 | 207 | 98349 | 26 | 476 |
| **Pneumonia** | 62 | 1764 | 198 | 97976 | 28 | 494 |
| **DASHI points** | | | | | | |
| 1 | 254 | 70598 | 6 | 29142 | 278 | 4686 |
| 2 | 240 | 47280 | 20 | 52460 | 197 | 2605 |
| 3 | 213 | 27190 | 48 | 72549 | 128 | 1528 |
| 4 | 172 | 14025 | 88 | 85714 | 81 | 972 |
| 5 | 132 | 6761 | 129 | 92979 | 51 | 721 |
| 6 | 84 | 2651 | 177 | 97089 | 32 | 549 |
| 7 | 51 | 1053 | 209 | 98686 | 20 | 473 |
| 8 | 37 | 647 | 223 | 99093 | 17 | 444 |
| 9 | 14 | 204 | 247 | 99356 | 15 | 403 |
| 10 | 2 | 27 | 259 | 99712 | 14 | 386 |
| 11 | <1 | 1 | 260 | 99738 | 17 | 383 |

Table S5: Diagnostic performance measures for DASHI score over the range of possible points

| **DASHI Points Threshold** | **Sensitivity %**  **(95% CI)** | **Specificity %**  **(95% CI)** | **Positive likelihood ratio (95% CI)** | **Negative likelihood ratio**  **(95% CI)** | **PPV % (95% CI)** | **NPV % (95% CI)** |
| --- | --- | --- | --- | --- | --- | --- |
| 1 | 97.74 (96.08 to 99.39) | 27.74 (27.64 to 27.85) | 1.35 (1.35 to 1.36) | NC | 0.35 (0.34 to 0.36) | 99.98 (99.98 to 99.98) |
| 2 | 92.33 (90.73 to 94.95) | 52.16 (52.07 to 52.24) | 1.93 (1.92 to 1.94) | 0.15 (0.06 to 0.23) | 0.50 (0.49 to 0.51) | 99.96 (99.96 to 99.96) |
| 3 | 82.21 (80.68 to 83.75) | 72.12 (72.06 to 72.19) | 2.95 (2.93 to 2.96) | 0.25 (0.20 to 0.30) | 0.76 (0.74 to 0.78) | 99.94 (99.93 to 99.94) |
| 4 | 66.54 (65.16 to 67.91) | 85.54 (85.49 to 85.59) | 4.60 (4.58 to 4.63) | 0.39 (0.36 to 0.42) | 1.19 (1.15 to 1.22) | 99.90 (99.89 to 99.90) |
| 5 | 50.80 (49.57 to 52.03) | 93.10 (93.07 to 93.13) | 7.36 (7.30 to 7.42) | 0.53 (0.50 to 0.55) | 1.89 (1.82 to 1.95) | 99.86 (99.86 to 99.87) |
| 6 | 33.01 (32.00 to 34.01) | 97.18 (97.16 to 97.20) | 11.71 (11.55 to 11.86) | 0.68 (0.67 to 0.71) | 2.97 (2.84 to 3.09) | 99.82 (99.82 to 99.83) |
| 7 | 20.08 (19.30 to 20.86) | 98.90 (98.89 to 98.92) | 18.33 (18.04 to 18.63) | 0.80 (0.80 to 0.82) | 4.57 (4.33 to 4.81) | 99.79 (99.78 to 99.80) |
| 8 | 14.32 (13.68 to 15.00) | 99.35 (99.34 to 99.36) | 22.00 (21.77 to 22.23) | 0.86 (0.85 to 0.87) | 5.43 (5.10 to 5.77) | 99.78 (99.77 to 99.78) |
| 9 | 5.81 (5.40 to 6.23) | 99.77 (99.77 to 99.78) | 25.66 (25.17 to 26.14) | 0.94 (0.94 to 0.95) | 6.28 (5.67 to 6.88) | 99.75 (99.74 to 99.75) |
| 10 | 0.84 (0.63 to 1.04) | 99.97 (99.96 to 99.97) | 26.83 (20.56 to 33.10) | 0.99 (0.98 to 0.99) | 6.55 (4.58 to 8.52) | 99.74 (99.74 to 99.75) |
| 11 | 0.03 (0.006 to 0.06) | 99.99 (99.99 to 1.00) | 19.19 (16.08 to 22.31) | 0.99 (0.99 to 1.00) | NC | 99.74 (99.73 to 99.75) |
| Diagnostic performance of DASHI score for primary CVD events in the 28 days following presentation with respiratory infection. Estimates derived in the external calibration dataset. DASHI points threshold = this number of points or more on the DASHI score. NC: estimates not calculable due to low numbers. | | | | | | |

Supplementary discussion

Table S5 demonstrates the trade-off between true to false positives and negatives. To use a DASHI score of four as a threshold would mean treating 81 false positives to treat a single true positive, and not treating 972 patients would miss treating one who went on to have a CVD event. These numbers reflect the context. Low prevalence of serious disease is the general problem in primary care where outcomes are rare, but the sheer number of low-risk people means that in aggregate the outcomes are important.[14] Primary care mostly only has low probabilities of harm because almost everything serious is rare, from screening for cervical cancers (1:2000 plus) to identifying febrile children who go on to have sepsis (1:444).[14,15]

Tools are one strategy to help deal with risk, and it may be that they can never achieve flattering ratios of false positive to true positives.[14] However, we can see that this performance is still better than the status quo, which is to assume no-one has any risk of CVD events from their RTI as current primary care guidelines don’t address this risk.[16]

Table S5 demonstrates the trade-off between sensitivity and specificity. Sensitivity is very high for one DASHI point (97.74%, 95% CI 96.08 to 99.39), but with very low specificity (27.74%, 95% CI 27.64 to 27.85). At ten DASHI points sensitivity reduces to less than one percent (0.84%, 95% CI 0.63 to 1.04) and specificity is very high at 99.97 (95% CI 99.96 to 99.97). Because the outcome is relatively rare negative likelihood ratios are all low and negative predictive values are very high (>99%) for all the possible scores. Positive predictive values range from 0.35% (95% 0.34% to 0.36%) for one point to 6.55% (95% CI 4.58% to 8.52%) for ten points.

## RECORD Checklist

**The RECORD statement – checklist of items, extended from the STROBE statement, that should be reported in observational studies using routinely collected health data.**

|  | **Item No.** | **STROBE items** | **Location in manuscript where items are reported** | **RECORD items** | **Location in manuscript where items are reported** |
| --- | --- | --- | --- | --- | --- |
| **Title and abstract** | | | | | |
|  | 1 | (a) Indicate the study’s design with a commonly used term in the title or the abstract (b) Provide in the abstract an informative and balanced summary of what was done and what was found | Title and abstract | RECORD 1.1: The type of data used should be specified in the title or abstract. When possible, the name of the databases used should be included.  RECORD 1.2: If applicable, the geographic region and timeframe within which the study took place should be reported in the title or abstract.  RECORD 1.3: If linkage between databases was conducted for the study, this should be clearly stated in the title or abstract. | Title and abstract  Abstract  Abstract |
| **Introduction** | | | | | |
| Background rationale | 2 | Explain the scientific background and rationale for the investigation being reported | Introduction |  |  |
| Objectives | 3 | State specific objectives, including any prespecified hypotheses | Methods: Objectives |  |  |
| **Methods** | | | | | |
| Study Design | 4 | Present key elements of study design early in the paper | Methods, Study design and setting |  |  |
| Setting | 5 | Describe the setting, locations, and relevant dates, including periods of recruitment, exposure, follow-up, and data collection | Methods, Study design and setting |  |  |
| Participants | 6 | *(a) Cohort study* - Give the eligibility criteria, and the sources and methods of selection of participants. Describe methods of follow-up  *Case-control study* - Give the eligibility criteria, and the sources and methods of case ascertainment and control selection. Give the rationale for the choice of cases and controls  *Cross-sectional study* - Give the eligibility criteria, and the sources and methods of selection of participants  *(b) Cohort study* - For matched studies, give matching criteria and number of exposed and unexposed  *Case-control study* - For matched studies, give matching criteria and the number of controls per case | Methods, Population paragraph  Neither matched, nor case-control | RECORD 6.1: The methods of study population selection (such as codes or algorithms used to identify subjects) should be listed in detail. If this is not possible, an explanation should be provided.  RECORD 6.2: Any validation studies of the codes or algorithms used to select the population should be referenced. If validation was conducted for this study and not published elsewhere, detailed methods and results should be provided.  RECORD 6.3: If the study involved linkage of databases, consider use of a flow diagram or other graphical display to demonstrate the data linkage process, including the number of individuals with linked data at each stage. | Supplementary materials, Github.  n/a  Supplementary materials |
| Variables | 7 | Clearly define all outcomes, exposures, predictors, potential confounders, and effect modifiers. Give diagnostic criteria, if applicable. | Methods and Supplementary methods | RECORD 7.1: A complete list of codes and algorithms used to classify exposures, outcomes, confounders, and effect modifiers should be provided. If these cannot be reported, an explanation should be provided. | Supplementary materials for ICD codes, OPCS codes, and CPRD search strategy available from JJL |
| Data sources/ measurement | 8 | For each variable of interest, give sources of data and details of methods of assessment (measurement).  Describe comparability of assessment methods if there is more than one group | Methods (more detail given in supplementary methods) |  |  |
| Bias | 9 | Describe any efforts to address potential sources of bias | Methods, population |  |  |
| Study size | 10 | Explain how the study size was arrived at | Methods, sample size |  |  |
| Quantitative variables | 11 | Explain how quantitative variables were handled in the analyses. If applicable, describe which groupings were chosen, and why | Methods, supplementary methods |  |  |
| Statistical methods | 12 | (a) Describe all statistical methods, including those used to control for confounding  (b) Describe any methods used to examine subgroups and interactions  (c) Explain how missing data were addressed  (d) *Cohort study* - If applicable, explain how loss to follow-up was addressed  *Case-control study* - If applicable, explain how matching of cases and controls was addressed  *Cross-sectional study* - If applicable, describe analytical methods taking account of sampling strategy  (e) Describe any sensitivity analyses | Methods, supplementary methods |  |  |
| Data access and cleaning methods |  | .. |  | RECORD 12.1: Authors should describe the extent to which the investigators had access to the database population used to create the study population.  RECORD 12.2: Authors should provide information on the data cleaning methods used in the study. | Supplementary materials  N/a |
| Linkage |  | .. |  | RECORD 12.3: State whether the study included person-level, institutional-level, or other data linkage across two or more databases. The methods of linkage and methods of linkage quality evaluation should be provided. | Supplementary methods: data linkages |
| **Results** | | | | | |
| Participants | 13 | (a) Report the numbers of individuals at each stage of the study (*e.g.*, numbers potentially eligible, examined for eligibility, confirmed eligible, included in the study, completing follow-up, and analysed)  (b) Give reasons for non-participation at each stage.  (c) Consider use of a flow diagram | Results, table 1 | RECORD 13.1: Describe in detail the selection of the persons included in the study (*i.e.,* study population selection) including filtering based on data quality, data availability and linkage. The selection of included persons can be described in the text and/or by means of the study flow diagram. | Methods, sup methods and figure S1 |
| Descriptive data | 14 | (a) Give characteristics of study participants (*e.g.*, demographic, clinical, social) and information on exposures and potential confounders  (b) Indicate the number of participants with missing data for each variable of interest  (c) *Cohort study* - summarise follow-up time (*e.g.*, average and total amount) | Results, table 1  Results, table 1  Methods |  |  |
| Outcome data | 15 | *Cohort study* - Report numbers of outcome events or summary measures over time  *Case-control study* - Report numbers in each exposure category, or summary measures of exposure  *Cross-sectional study* - Report numbers of outcome events or summary measures | Results, table 1 |  |  |
| Main results | 16 | (a) Give unadjusted estimates and, if applicable, confounder-adjusted estimates and their precision (e.g., 95% confidence interval). Make clear which confounders were adjusted for and why they were included  (b) Report category boundaries when continuous variables were categorized  (c) If relevant, consider translating estimates of relative risk into absolute risk for a meaningful time period | n/a - prediction  Methods and sup methods  n/a |  |  |
| Other analyses | 17 | Report other analyses done—e.g., analyses of subgroups and interactions, and sensitivity analyses | Results, Supp results |  |  |
| **Discussion** | | | | | |
| Key results | 18 | Summarise key results with reference to study objectives | Discussion, principle findings |  |  |
| Limitations | 19 | Discuss limitations of the study, taking into account sources of potential bias or imprecision. Discuss both direction and magnitude of any potential bias | Discussion, Strengths and limitations | RECORD 19.1: Discuss the implications of using data that were not created or collected to answer the specific research question(s). Include discussion of misclassification bias, unmeasured confounding, missing data, and changing eligibility over time, as they pertain to the study being reported. | Discussion, Strengths and limitations |
| Interpretation | 20 | Give a cautious overall interpretation of results considering objectives, limitations, multiplicity of analyses, results from similar studies, and other relevant evidence | Discussion, Implications for practice and research |  |  |
| Generalisability | 21 | Discuss the generalisability (external validity) of the study results | Discussion, Implications for practice and research |  |  |
| **Other Information** | | | | | |
| Funding | 22 | Give the source of funding and the role of the funders for the present study and, if applicable, for the original study on which the present article is based | Funding and rights retention |  |  |
| Accessibility of protocol, raw data, and programming code |  | .. | Methods, supplementary methods | RECORD 22.1: Authors should provide information on how to access any supplemental information such as the study protocol, raw data, or programming code. | Supplementary materials |

*Reference: Benchimol EI, Smeeth L, Guttmann A, Harron K, Moher D, Petersen I, Sørensen HT, von Elm E, Langan SM, the RECORD Working Committee. The REporting of studies Conducted using Observational Routinely-collected health Data (RECORD) Statement. *PLoS Medicine* 2015; in press.

*Checklist is protected under Creative Commons Attribution ([CC BY](http://creativecommons.org/licenses/by/4.0/)) license.

Tripod Checklist

| **Section/Topic** | **Item** |  | **Checklist Item** | **Page** |
| --- | --- | --- | --- | --- |
| **Title and abstract** | | | | |
| Title | 1 | D;V | Identify the study as developing and/or validating a multivariable prediction model, the target population, and the outcome to be predicted. | 1 |
| Abstract | 2 | D;V | Provide a summary of objectives, study design, setting, participants, sample size, predictors, outcome, statistical analysis, results, and conclusions. | 4 |
| **Introduction** | | | | |
| Background and objectives | 3a | D;V | Explain the medical context (including whether diagnostic or prognostic) and rationale for developing or validating the multivariable prediction model, including references to existing models. | 6 |
|  | 3b | D;V | Specify the objectives, including whether the study describes the development or validation of the model or both. | 6 |
| **Methods** | | | | |
| Source of data | 4a | D;V | Describe the study design or source of data (e.g., randomized trial, cohort, or registry data), separately for the development and validation data sets, if applicable. | 7 |
|  | 4b | D;V | Specify the key study dates, including start of accrual; end of accrual; and, if applicable, end of follow-up. | 7 |
| Participants | 5a | D;V | Specify key elements of the study setting (e.g., primary care, secondary care, general population) including number and location of centres. | 7 |
|  | 5b | D;V | Describe eligibility criteria for participants. | 7 |
|  | 5c | D;V | Give details of treatments received, if relevant. | na |
| Outcome | 6a | D;V | Clearly define the outcome that is predicted by the prediction model, including how and when assessed. | 7 |
|  | 6b | D;V | Report any actions to blind assessment of the outcome to be predicted. | na |
| Predictors | 7a | D;V | Clearly define all predictors used in developing or validating the multivariable prediction model, including how and when they were measured. | 7/supp/online |
|  | 7b | D;V | Report any actions to blind assessment of predictors for the outcome and other predictors. | na |
| Sample size | 8 | D;V | Explain how the study size was arrived at. | 8 |
| Missing data | 9 | D;V | Describe how missing data were handled (e.g., complete-case analysis, single imputation, multiple imputation) with details of any imputation method. | 8 |
| Statistical analysis methods | 10a | D | Describe how predictors were handled in the analyses. | 8 |
|  | 10b | D | Specify type of model, all model-building procedures (including any predictor selection), and method for internal validation. | 8/supp |
|  | 10c | V | For validation, describe how the predictions were calculated. | 9 |
|  | 10d | D;V | Specify all measures used to assess model performance and, if relevant, to compare multiple models. | 8-9 |
|  | 10e | V | Describe any model updating (e.g., recalibration) arising from the validation, if done. | na |
| Risk groups | 11 | D;V | Provide details on how risk groups were created, if done. | na |
| Development vs. validation | 12 | V | For validation, identify any differences from the development data in setting, eligibility criteria, outcome, and predictors. | 8 |
| **Results** | | | | |
| Participants | 13a | D;V | Describe the flow of participants through the study, including the number of participants with and without the outcome and, if applicable, a summary of the follow-up time. A diagram may be helpful. | 9/supp |
|  | 13b | D;V | Describe the characteristics of the participants (basic demographics, clinical features, available predictors), including the number of participants with missing data for predictors and outcome. | T1 |
|  | 13c | V | For validation, show a comparison with the development data of the distribution of important variables (demographics, predictors and outcome). | T1 |
| Model development | 14a | D | Specify the number of participants and outcome events in each analysis. | T1 |
|  | 14b | D | If done, report the unadjusted association between each candidate predictor and outcome. | Na |
| Model specification | 15a | D | Present the full prediction model to allow predictions for individuals (i.e., all regression coefficients, and model intercept or baseline survival at a given time point). | Supp |
|  | 15b | D | Explain how to the use the prediction model. | 12 |
| Model performance | 16 | D;V | Report performance measures (with CIs) for the prediction model. | T3 |
| Model-updating | 17 | V | If done, report the results from any model updating (i.e., model specification, model performance). | NA |
| **Discussion** | | | | |
| Limitations | 18 | D;V | Discuss any limitations of the study (such as nonrepresentative sample, few events per predictor, missing data). | 11 |
| Interpretation | 19a | V | For validation, discuss the results with reference to performance in the development data, and any other validation data. | 10 |
|  | 19b | D;V | Give an overall interpretation of the results, considering objectives, limitations, results from similar studies, and other relevant evidence. | 10 |
| Implications | 20 | D;V | Discuss the potential clinical use of the model and implications for future research. | 12 |
| **Other information** | | | | |
| Supplementary information | 21 | D;V | Provide information about the availability of supplementary resources, such as study protocol, Web calculator, and data sets. | Supp/online |
| Funding | 22 | D;V | Give the source of funding and the role of the funders for the present study. | 13 |

*Items relevant only to the development of a prediction model are denoted by D, items relating solely to a validation of a prediction model are denoted by V, and items relating to both are denoted D;V. We recommend using the TRIPOD Checklist in conjunction with the TRIPOD Explanation and Elaboration document.

Supplementary references

1 Wolf A, Dedman D, Campbell J, *et al.* Data resource profile: Clinical Practice Research Datalink (CPRD) Aurum. *Int J Epidemiol*. 2019;48:1740-1740G. doi: 10.1093/ije/dyz034

2 Herrett E, Gallagher AM, Bhaskaran K, *et al.* Data Resource Profile: Clinical Practice Research Datalink (CPRD). *Int J Epidemiol*. 2015;44:827–36. doi: 10.1093/ije/dyv098

3 GOV.UK. The English Indices of Deprivation 2019- Statistical Release. *Ministry of Housing, Communities and Local Government*. 2019;2019:1–12.

4 Hippisley-Cox J, Coupland C, Brindle P. Development and validation of QRISK3 risk prediction algorithms to estimate future risk of cardiovascular disease: Prospective cohort study. *BMJ (Online)*. 2017;357:1–21. doi: 10.1136/bmj.j2099

5 Smeeth L, Thomas SL, Hall AJ, *et al.* Risk of Myocardial Infarction and Stroke after Acute Infection or Vaccination. *New England Journal of Medicine*. 2004;351:2611–8. doi: 10.1056/NEJMoa041747

6 Sharma A, Pagidipati NJ, Califf RM, *et al.* Impact of Regulatory Guidance on Evaluating Cardiovascular Risk of New Glucose-Lowering Therapies to Treat Type 2 Diabetes Mellitus: Lessons Learned and Future Directions. *Circulation*. 2020;141:843–62. doi: 10.1161/CIRCULATIONAHA.119.041022

7 Davidson JA, Banerjee A, Muzambi R, *et al.* Validity of acute cardiovascular outcome diagnoses in European electronic health records: A systematic review protocol. *BMJ Open*. 2019;9:1095–111. doi: 10.1136/bmjopen-2019-031373

8 CPRD. CPRD Aurum Frequently asked questions (FAQs). 2021.

9 Ebell MH, Afonso A. A Systematic Review of Clinical Decision Rules for the Diagnosis of Influenza. *Ann Fam Med*. 2011;9:69–77. doi: 10.1370/afm.1192.INTRODUCTION

10 Ramsay M (UK HSA. *The Green Book*. 2023.

11 Hamilton F, Arnold D, Henley W, *et al.* Aspirin reduces cardiovascular events in patients with pneumonia: a prior event rate ratio analysis in a large primary care database. *European Respiratory Journal*. 2020;2002795. doi: 10.1183/13993003.02795-2020

12 Riley RD, Snell KIE, Ensor J, *et al.* Minimum sample size for developing a multivariable prediction model: PART II - binary and time-to-event outcomes. *Stat Med*. 2019;38:1276–96. doi: 10.1002/sim.7992

13 Riley RD, Snell KIE, Archer L, *et al.* Evaluation of clinical prediction models (part 3): calculating the sample size required for an external validation study. *Bmj*. Published Online First: 2024. doi: 10.1136/bmj-2023-074821

14 Buntinx F, Mant D, Van Den Bruel A, *et al.* Dealing with low-incidence serious diseases in general practice. *British Journal of General Practice*. 2011;61:43–6. doi: 10.3399/bjgp11X548974

15 Van den Bruel A, Haj-Hassan T, Thompson M, *et al.* Diagnostic value of clinical features at presentation to identify serious infection in children in developed countries: a systematic review. *The Lancet*. 2010;375:834–45. doi: 10.1016/S0140-6736(09)62000-6

16 NICE. Respiratory tract infections (self-limiting): prescribing antibiotics | Guidance and guidelines | NICE. *NICE Guidelines*. 2008;20.

1. In order of expert ranking: Age, heart failure, diabetes, smoking status, renal disease, hypertension, peripheral vascular disease, COPD, systolic and diastolic BP, sex, total cholesterol to HDL cholesterol ratio, BMI, Atrial arrhythmia, dementia, anticoagulation, non-steroidal anti-inflammatory prescriptions, obstructive sleep apnoea, antiplatelet use, antihypertensives, statins, rheumatoid arthritis, platelets, CRP, erectile dysfunction, learning disability, chronic or congenital heart disease, IMD score, cancers (solid and haematological), family history of CVD, pneumococcal vaccine, glucose lowering medications, chemotherapy in the last 6 months, influenza vaccine, white cell count, haemoglobin, lupus, asthma, antibiotics on the day of consultation, splenic dysfunction, immunosuppression, connective tissue disorders, ethnicity, alcohol consumption (moderate or heavy), other chronic lung disease, chronic liver disease, migraine, coeliac disease, drugs that cause bleeding (from BNF), recent cellulitis, seizure disorders, other chronic neurological conditions, UTI number in last 5 years. [↑](#footnote-ref-1)
